# Generation and Application of a Reporter Cell Line for the Quantitative Screen of Extracellular Vesicle Release

**DOI:** 10.3389/fphar.2021.668609

**Published:** 2021-04-16

**Authors:** Jonathan Shpigelman, Fitzgerald S. Lao, Shiyin Yao, Chenyang Li, Tetsuya Saito, Fumi Sato-Kaneko, John P. Nolan, Nikunj M. Shukla, Minya Pu, Karen Messer, Howard B. Cottam, Dennis A. Carson, Maripat Corr, Tomoko Hayashi

**Affiliations:** ^1^ Moores Cancer Center, University of California, San Diego, La Jolla, CA, United States; ^2^ School of Pharmaceutical Sciences, Health Science Center, Shenzhen University, Shenzhen, China; ^3^ Department of Rheumatology, Graduate School of Medical and Dental Sciences, Tokyo Medical and Dental University, Tokyo, Japan; ^4^ Scintillon Institute, San Diego, CA, United States; ^5^ Division of Biostatistics and Bioinformatics, Department of Family Medicine and Public Health, University of California, San Diego, La Jolla, CA, United States; ^6^ Department of Medicine, University of California, San Diego, La Jolla, CA, United States

**Keywords:** extracellular vesicle, exosome, THP-1, CD63, CD9, luciferase, antigen-presenting cells

## Abstract

Extracellular vesicles (EVs) are identified as mediators of intercellular communication and cellular regulation. In the immune system, EVs play a role in antigen presentation as a part of cellular communication. To enable drug discovery and characterization of compounds that affect EV biogenesis, function, and release in immune cells, we developed and characterized a reporter cell line that allows the quantitation of EVs shed into culture media in phenotypic high-throughput screen (HTS) format. Tetraspanins CD63 and CD9 were previously reported to be enriched in EVs; hence, a construct with dual reporters consisting of CD63-Turbo-luciferase (Tluc) and CD9-Emerald green fluorescent protein (EmGFP) was engineered. This construct was transduced into the human monocytic leukemia cell line, THP-1. Cells expressing the highest EmGFP were sorted by flow cytometry as single cell, and clonal pools were expanded under antibiotic selection pressure. After four passages, the green fluorescence dimmed, and EV biogenesis was then tracked by luciferase activity in culture supernatants. The Tluc activities of EVs shed from CD63Tluc-CD9EmGFP reporter cells in the culture supernatant positively correlated with the concentrations of released EVs measured by nanoparticle tracking analysis. To examine the potential for use in HTS, we first miniaturized the assay into a robotic 384-well plate format. A 2210 commercial compound library (Maybridge) was then screened twice on separate days, for the induction of extracellular luciferase activity. The screening data showed high reproducibility on days 1 and 2 (78.6%), a wide signal window, and an excellent Z′ factor (average of 2-day screen, 0.54). One hundred eighty-seven compounds showed a response ratio that was 3SD above the negative controls in both day 1 and 2 screens and were considered as hit candidates (approximately 10%). Twenty-two out of 40 re-tested compounds were validated. These results indicate that the performance of CD63Tluc-CD9EmGFP reporter cells is reliable, reproducible, robust, and feasible for HTS of compounds that regulate EV release by the immune cells.

## Introduction

Cells generate small vesicles by inward or outward budding of multivesicular bodies or late endosomes and release them into extracellular spaces (Woodman and Futter, 2008; Gireud-Goss et al., 2018). These extracellular vesicles (EVs) are produced by multiple cell types and include exosomes, microvesicles, and oncosomes (Stahl and Raposo, 2018). They are found abundantly in body fluids, including saliva, blood, urine, and breast milk, presumably as means of disposal or dissemination from their source to distal targets (Zwicker et al., 2009; Yanez-Mo et al., 2015; Foster et al., 2016). EVs are thought to play an essential role in cell-to-cell communication as they carry cell-type-specific molecules, including those involved in innate immune responses, such as cytokines, chemokines, intercellular adhesion molecules (ICAMs), coding and non-coding RNAs (including microRNAs), lipids, proteins, peptides and DNA fragments (Valadi et al., 2007; Skog et al., 2008; Cossetti et al., 2014; Yanez-Mo et al., 2015). Certain molecules integrated into the EV outer surface membrane direct adhesion to potential target cells, and their cargos can confer specific intercellular communications. These properties enable EVs to play modulating roles in mediating immune responses to pathogens and tumors (Campos et al., 2015; Wang et al., 2017). Chemically controlled EV biogenesis could lead to novel immunomodulation modalities, but the mechanisms that control cargo loading and EV formation are yet to be fully defined. Quantifying EVs’ cellular biogenesis in small samples would enable chemical screening to define pathways involved in EV production and release.

In immune responses, EVs have been reported to play immune-stimulatory and suppressive roles. We previously reported that tumor cells deficient in large tumor suppressor kinase (LATS)1/2 (part of the Hippo pathway) released immunostimulatory EVs carrying tumor-specific antigens and RNA (Moroishi et al., 2016). Such EVs from the LATS1/2 deficient tumor cell lines induced tumor-specific adaptive immune responses (Moroishi et al., 2016). EVs from antigen-presenting cells (APCs) have essential biological functions, as EVs released from dendritic cells (DCs) could stimulate pro-inflammatory responses and induce antigen-specific T cell responses (Zitvogel et al., 1998; Escudier et al., 2005; Morse et al., 2005; Besse et al., 2016). Pooled EVs shed from mature DCs expressing CD54, CD86, and MHC-class-I and II molecules effectively stimulated T cells (Wen et al., 2017). In contrast to immune-stimulating EVs, EVs can also act as immune suppressors. EVs from tumor cells suppressed tumor-specific immune responses by inhibiting APC maturation, inducing monocyte-derived suppressor cells and regulatory T cells (Treg), and promoting apoptosis of activated cytotoxic T lymphocytes (Taylor and Gercel-Taylor, 2011; Lindenbergh and Stoorvogel, 2018).

High throughput screening (HTS) is indispensable for the current drug discovery process (Macarron and Hertzberg, 2011). Compounds that regulate EV release with various immunostimulatory or antigen-presenting properties would be potential candidates for cancer immune therapy or vaccine adjuvants for infectious diseases. Thus, phenotypic HTS technology quantifying EV release by immune reporter cells could be an essential initial step for drug development for immune related conditions. Reporter cells amenable for phenotypic HTS need to not only be relevant for the biological activities in question, but also robust and reproducible in miniaturized 384- or 1556-well formats (Macarron and Hertzberg, 2011). Although a few reports have utilized tetraspanins (TSs) to monitor or label EVs in the culture supernatant, these reporter cells used non-immune cells and were not optimized for the HTS platform (Hikita et al., 2018; Cashikar and Hanson, 2019; Gupta et al., 2020). Thus, this study aimed to develop a stable human monocytic reporter cell line (THP-1 cells) that could quantitatively measure EV release and screen for compounds that alter EV release levels from innate immune cells. To monitor EV release into culture supernatants, we employed TSs (CD9 and CD63) for the reporter construct (Andreu and Yanez-Mo, 2014; Kowal et al., 2016). The reporter cells were characterized and then used in a 384-well format robotic screen of over 2000 compounds. The data demonstrated the robustness and reproducibility of the CD63Tluc-CD9EmGFP THP-1 reporter cells for screening compounds that modulate EV release by the immune cells.

## Materials and Methods

### Generation of CD63Tluc-CD9EmGFP Lentivirus

A synthetic gene sequence encoding CD63 fused to the Turbo luciferase gene and then linked to an internal ribosomal entry site (IRES) and CD9 encoding sequences fused to that for emerald green fluorescent protein (EmGFP, derived from *Aequorea Victoria* GFP) was assembled from synthetic oligonucleotides and/or PCR products (Supplementary Figure S1A). The fragment was inserted into the lentiviral vector pLenti6.3_V5-DEST_A244 (Thermo Fisher Scientific, Waltham, MA, United States) according to the manufacturer's instructions. Plasmid DNA was purified from transformed *Escherichia coli*, and the concentration was determined by UV spectroscopy. The sequence in the insertion sites in the final construct was verified using next-generation sequencing technology and was verified to be accurate. A map of the final construct (pLenti CD63Tluc-CD9EmGFP) is shown in Supplementary Figure S1B. Lentivirus was generated using a proprietary high titer yield protocol at Thermo Fisher Scientific. Briefly, HEK293T cells were transfected with the pLenti CD63-TlucCD9-EmGFP vector using ViraPower™ Lentiviral Packaging Mix (K497500, Thermo Fisher Scientific) and Lipofectamine™ 3000 (L3000075, Thermo Fisher Scientific). After 48 h, the released lentivirus was collected from the conditioned medium. The lentiviral preparation was concentrated ten-fold and the viral titer was determined using Lenti-X™GoStix™ (#631280, Takara Bio United States Inc., Mountain View, CA, United States).

### Transduction, Cloning and Selection of THP-1 Cells Stably Expressing CD63 Turbo-Luciferase and CD9-EmGFP

THP-1 cells were cultured in RPMI 1640 medium supplemented with 10% dialyzed FBS (S12850H, Atlanta Biologicals), 100 U/ml penicillin, 100 μg/ml streptomycin (15140122, Gibco, Thermo Fisher Scientific), 1 mM sodium pyruvate (11360-070, Gibco, Thermo Fisher Scientific) and 1 × MEM non-essential amino acids (11140-050, Gibco, Thermo Fisher Scientific) at 37°C in 5% CO_2_. THP-1 cells were transduced with lentivirus encoding CD63Tluc-CD9EmGFP at varying dilutions (1:2, 1:5, 1:10, 1:50, 1:250 and 1:500) in the presence of polybrene (8 μg/ml). After transduction, the culture was centrifuged at 2,500 rpm for 30 min to improve the transduction efficiency. The antibiotic-resistant cells were collected and analyzed for EmGFP expression by flow cytometry. Tluc activity was measured using the TurboLuc™ Luciferase One-Step Glow Assay Kit (88264, Thermo Fisher Scientific). A stable pool of cells expressing CD63Tluc and CD9EmGFP was expanded in RPMI containing 5 μg/ml blasticidin (Gibco Thermo Fisher Scientific). Single EmGFP^hi^ cells were sorted by flow cytometry into individual wells of six 96-well plates to establish clones. EmGFP expression was validated by flow cytometry and Tluc expression was verified by the TurboLuc™ Luciferase One-Step Glow Assay Kit as described above.

### Culture and Treatment of CD63 Turbo-Luciferase-CD9EmGFP Reporter Cells and Parental THP-1 Cells

The selected clone (designated as CD63Tluc-CD9EmGFP onward) was cultured in HEPES buffered RPMI 1640 medium (72400, Thermo Fisher Scientific) supplemented with 10% dialyzed FBS, 100 U/ml penicillin, 100 μg/ml streptomycin, 1 mM sodium pyruvate, 1 × MEM non-essential amino acids, and 5 μg/ml blasticidin. Parental THP-1 cells were cultured in RPMI 1640 medium (11875, Thermo Fisher Scientific) supplemented with 10% FBS, 100 U/ml penicillin, 100 μg/ml streptomycin, and 50 µM 2-mercaptoethanol. Both cell types were maintained in humidified conditions with 5% CO_2_ at 37°C.

Prior to treatment, CD63Tluc-CD9EmGFP reporter cells and parental THP-1 cells were washed with their base medium and re-suspended in their complete culture medium substituted with exosome-depleted FBS (EXO-FBS-250A-1, System Biosciences, Mountain View, CA, United States, or Gibco™ Exosome-Depleted FBS, Thermo Fisher Scientific). In the experiments performed for Figures 2A–G (Thermo Fisher), CD63Tluc-CD9EmGFP reporter cells were treated with graded concentrations of phorbol myristate acetate (PMA) (tlrl-pma, InvivoGen, San Diego, CA, United States) or immunostimulatory compounds (5 and 81) (Sato-Kaneko et al., 2018; Chan et al., 2019). Cell concentrations were 5 × 10^4^ cells/200 µl/well for 96-well format and 2.5 × 10^4^ cells/80 µl/well for 384-well format unless otherwise indicated in the figure legends. In the experiments performed for Figures 2H, 3, and 4, and Tables 1, 2, CD63Tluc-CD9EmGFP reporter cells and parental THP-1 cells were treated at 2.5 or 5 × 10^5^ cells/ml in 6-, 96 well tissue culture plates or flasks with 10 ng/ml lipopolysaccharide (LPS, Ultrapure LPS, tlrl-3pelps, InvivoGen), 50 ng/ml or graded concentrations of PMA, 5 µM compound 5, 5 µM compound 81, 5 µM GW4869 (GW, 13127, Cayman Chemical Company, Ann Arbor, MI, United States) or 1 µM manumycin A (MA, 10010497, Cayman Chemical Company). All treatments were performed for 48 h in humidified conditions with 5% CO_2_ at 37°C.

**TABLE 1:** Tluc activity in CD63TlucCD9EmGFP reporter cells and culture supernatants.

Treatment	Cells (×10^3^)^a^	Supernatant (×10^3^)^a^ ^,^ ^b^	% Release^a^ ^,^ ^b^
Vehicle	37.3 ± 19.4	1.03 ± 0.06	3.6 ± 2.2
LPS	138.9 ± 97.6	2.59 ± 0.9^d^	2.4 ± 1
PMA	201.2 ± 75.1^c^	3.66 ± 0.62^d^	2.0 ± 0.5
#5	86.2 ± 23.8	3.12 ± 0.33^d^	3.8 ± 1.1
#81	85.5 ± 18.3	3.34 ± 0.41^d^	4.1 ± 1.1

^a^ mean ± SD of four independent experiments. The raw data were presented in the Supplementary Table S1.

^b^ % release = luciferase activity of supernatant/(Tluc activities of cells + supernatant).

^c^ *p* < 0.005 vs. vehicle treated conditiond.

^d^ *p* ≦ 0.0001 vs. vehicle treated condition.

**TABLE 2:** Profiles of EVs released from CD63TlucCD9EmGFP reporter cells and parental THP-1 cells.

	CD63TlucCD9GFP reporter cells			Parental THP-1 cells		
Samples	Total protein (µg)/10^6^ cells^a^	Particles (× 10^9^/ml)/10^6^ cells^a^	Mean size (nm)^a^	Total protein (µg)/10^6^ cells^a^	Particles (× 10^9^/ml)/10^6^ cells^a^	Mean size (nm)^a^
Vehicle	18.1 ± 0.88	1.5 ± 0.07	161.0 ± 0.7	11.6 ± 0.35	1.4 ± 0.25	160.8 ± 2.7
PMA (50 ng/ml)	18.5 ± 1.5	2.0 ± 0.09^b^	148.7 ± 5.9	18.5 ± 1.8	2.0 ± 0.12^b^	156.1 ± 3.1

^a^ Mean ± SD of two independent experiments.

^b^ *p* < 0.05 by Student's t test vs. vehicle treated condition.

^c^ *p* < 0.0001 by Student's t test vs. vehicle treated condition.

### Extracellular Vesicle Isolation

Four EV isolation methods, Total exosome isolation reagent™, ExoQuick-TC™ exosome isolation reagent, polyethylene glycol (PEG) precipitation and differential ultracentrifugation, were used.

#### Total Exosome Isolation Reagent™

The culture plates were centrifuged at 2,000 *g* for 10 min. The supernatants were first mixed with an aliquot of Total Exosome Isolation Reagent (Thermo Fisher Scientific) equal to 1/2 the total volume of culture supernatant and then incubated at 4°C overnight. Following a 10,000 *g* spin for 1 h at 4°C, all traces of supernatant were discarded, and pellets were re-suspended in particle-free PBS.

#### ExoQuick-TC Exosome Isolation Reagent

After treatment, culture supernatants were spun at 250 g to remove cells. Supernatants were then filtered using a 0.45 µm syringe filter (MilliporeSigma, Sigma-Aldrich, St. Louis, MO, United States). Filtrates were then mixed with an aliquot of ExoQuick-TC Exosome Isolation Reagent (System Biosciences) equal to 1/5 the total volume of culture supernatant and incubated at 4°C overnight. Mixtures were then spun at 3,000 *g* for 15 min at 4°C, and an aliquot of this supernatant (EV-depleted fraction) was saved. Following another 3,000 *g* spin for 15 min at 4°C, all remaining traces of fluid were removed. Pellets were then resuspended in particle-free PBS.

#### Eight % PEG Precipitation

The culture plates were centrifuged at 300 *g* for 5 min. The supernatants were centrifuged at 2,000 *g* for 10 min at RT, and 160 µl supernatant were transferred to the new plates. An equal volume of cold 16% PEG/1 M NaCl (final 8% PEG) was mixed by pipetting, and the mixtures were incubated at 4°C overnight. The mixtures were centrifuged at 4,300 *g* at 4°C for 60 min. The supernatants were discarded, and pellets washed with 200 µl cold 5% PEG/1M NaCl/PBS (dilute 16% PEG/1 M NaCl with filtered PBS to final 5% PEG) at 4,300 *g* at 4°C for 15 min. The pellets were resuspended in particle-free PBS.

#### Differential Ultracentrifugation

The reporter cells or parental THP-1 cells were treated at a density of 5 × 10^5^/ml with vehicle or PMA (50 ng/ml) for 48 h in tissue culture flasks. Conditioned culture media (12 ml or 32 ml) was spun at 300 *g* for 10 min. Supernatants were subsequently spun at 2,000 *g* for another 10 min. Next, depending on volume, supernatants were transferred to 10 ml or 31.5 ml open-top polypropylene UC tubes (358120 and 358126, respectively, Beckman Coulter Life Sciences, CA, United States) and spun at 10,000*g*_avg_ for 30 min in an SW40Ti rotor (K-Factor: 2,771) or SW28 rotor (K-Factor: 2,554), respectively. 28.5 ml of supernatants were then transferred to new UC tubes and spun at 130,000*g*_avg_ (SW40Ti; K-Factor: 213) for 3 h or 100,000*g*_avg_ (SW28; K-Factor: 246) for 2 h by Beckman Optima XL-90 Ultracentrifuge (Beckman Coulter Life Sciences), respectively. The supernatants were then gently aspirated (leaving ∼50 µl), and pellets resuspended to their previous step volumes in cold PBS. Re-suspended pellets were then spun under the same conditions as their previous spin, followed by another round of gentle aspiration and resuspension to a final volume of either 100 µl (SW40Ti) or 150 µl (SW28) in cold PBS. All centrifugation steps were performed at 4°C, and resultant samples were stored at −80°C until use.

Additional details for each procedure are described in the figure legends.

### Measurement of Turbo-Luciferase Activity

Culture supernatant or isolated EV samples were plated in 96-well black wall, clear bottom assay plates (3603, Corning) or 384-well (164564, Thermo Fisher Scientific) and were allowed to equilibrate to RT. An equal volume of working Tluc assay reagent was added. The plate was incubated with agitation for 10 min at RT in the dark. Luminescence was then measured on the BMG CLARIOstar (BMGLabtech, Cary, NC) or TECAN Infinite M200 plate reader (Tecan, Mannedorf, Switzerland).

### Measurement of Extracellular Vesicle Concentrations and Size Distributions

Particle concentrations and size distributions were determined using nanoparticle tracking analysis (NTA) with a Nanosight NS300 instrument (Malvern Panalytical, Malvern, United Kingdoms) or microfluidic resistive pulse sensing with an nCS1 instrument (Spectradyne LLC, United States).

#### NanoSight NS300

Isolated EVs were diluted in particle-free PBS to the optimal concentration (10–150 particles per camera frame). A minimum of 700 µl of diluted sample was then manually injected using a 1-ml syringe before being installed on a syringe pump and infused at a rate of “100” for the duration of the video acquisitions. Three 60-s acquisitions of each sample were obtained and averaged for subsequent analysis. The camera level and detection threshold parameters in the NTA 3.1 software were maintained at “13” and “5”, respectively, for accurate and consistent measurement of particle concentration, mean diameter, and size distribution.

#### Spectradyne nCS1

Samples were analyzed using TS-400 cartridges for particles between 65 and 400 nm in diameter. Isolated EVs were diluted in particle-free PBS to the optimal cartridge particle concentration (10^7^–10^11^ particles/ml). 10-s measurements were performed for each sample at least 20 times and averaged. Unconditioned media without cells were measured for subtraction of background particles, including protein aggregates from exosome-depleted FBS. Mean size and particle concentrations were analyzed using the nCS1 Data Analyzer (Spectradyne). Only particles from 75 to 400 nm were included in the analysis to minimize the inclusion of false particle events.

### Electron Microscopy

EVs were isolated by ExoQuick-TC, resuspended to 1/500 original volume of culture supernatant, and stored at −80°C. For the physical characterization of EVs, negative stain transmission electron microscopy was performed as follows. Formvar-carbon-coated copper grids (100 mesh, Electron Microscopy Sciences, Hatfield, PA) were placed on 5 μl drops of each sample solution displayed on a parafilm sheet. After allowing the material to adhere to the grids for 10 min, grids were washed times times by rinsing through 200 μl drops of milli-Q water before being left for 1 min on 2% (wt/vol) uranyl acetate (Ladd Research Industries, Williston, VT) in water. Excess solution was removed with Whatman 3MM blotting paper, and grids were left to dry for a few minutes before viewing. Grids were examined using a Tecnai G2 Spirit BioTWIN transmission electron microscope equipped with an Eagle 4k HS digital camera (FEI, Hillsboro, OR, United States).

### Immunoblotting

For immunoblotting, EVs (30 µg protein) were mixed with 4 × NuPAGE sample buffer (NP0007, Thermo Fisher Scientific), with or without dithiothreitol (DTT) (D9779-5G, Sigma-Aldrich), and a protein inhibitor cocktail (Roche, Basel, Switzerland). The total protein in the samples was quantitated by Pierce BCA Protein Assay Kit. Samples were then mixed by pipetting and incubated on ice for 15 min. When DTT, a reducing agent, was used, samples were also denatured at 70°C for 10 min prior to loading. After fractionation on NuPAGE 4–12% Bis-Tris Gels (Thermo Fisher Scientific), samples were blotted onto Immobilon-P PVDF membranes (IPVH00010, Sigma) and blocked for 1 h in 5% BSA-TBS-T at RT. The blots were then incubated with primary antibodies (Ab): anti-CD63 Ab (1:1000 dilution) (sc-5275, Santa Cruz Biotechnology, Dallas, TX, United States), anti-CD81 Ab (1:500 dilution) (sc-23962; Santa Cruz Biotechnology), anti-Tsg101 Ab (1:500 dilution) (MA1-23296; Thermo Fisher Scientific), and anti-calnexin Ab (1:1000 dilution) (2679S; Cell Signaling, Danvers, MA, United States), overnight at 4°C with gentle agitation. After washing, the blots were incubated with anti-mouse secondary antibody (7076S: Cell Signaling) for blots probed against CD63, CD81, and Tsg101 or anti-rabbit secondary antibody (7074S: Cell Signaling) for blots probed against Calnexin for 30 min at RT with gentle agitation. Blots were developed with ProSignal Dura ECL Reagent (Prometheus Protein Biology Products, Genesee Scientific, San Diego, CA, United States) and visualized using a ChemiDoc Imaging System (Bio-Rad Laboratories, Hercules, CA, United States). Two molecular weight markers, AccuRuler Prestained Protein Ladder (G02101, Lamda Biotech, St. Louis, MO, United States) and Precision Plus Protein™ Dual Color Standards (1610374, Bio-Rad Laboratories) were used.

### Vesicle Flow Cytometry Assessing Extracellular Vesicle Number, Size and Composition

EV number, size and composition were determined by single vesicle flow cytometry (Stoner et al., 2016) using a commercial assay kit (vFC™ Assay kit, Cellarcus Biosciences, San Diego, CA, United States) and flow cytometer (CytoFlexS, Beckman Coulter, Indianapolis, IN, United States). Briefly, samples were stained with the fluorogenic membrane stain vFRed™ and, where indicated, carboxyfluorescein diacetate succinimidyl ester (CFSE, 40 μM, Cellarcus Biosciences) and antibodies against individual tetraspanins (Supplementary Table S2) or a mixture of antibodies against CD9, CD63, and CD81-labeled with phycoerythrin (PE, vTag™ anti-human TS PE mix, Cellarcus Biosciences) for 1 h at RT, diluted 1,000-fold in the buffer, and analyzed using membrane fluorescence to trigger detection. Data were analyzed using FCS Express, and included calibration using a vesicle size and fluorescence intensity standards. The analysis included a pre-stain dilution series in determining the optimal initial sample dilution and multiple positive and negative controls, as recommended by guidelines from the International Society for Extracellular Vesicles (ISEV) (Thery et al., 2018). A detailed description of vFC™ methods and controls are presented in Supplementary Information.

### Cell Viability Assay by 3- [4,5-Dimethylthiazol-2-yl]-2,5-dipheyl Tetrazolium Bromide (MTT)

0.5 mg/ml MTT (Thermo Fisher Scientific) solution was added to cell pellets in each well and incubated 6 h and formazan crystals were then lysed with lysis buffer (15% SDS and 0.12% 12 N HCl). The absorbance was measured at 570 nm using 650 nm as a reference with a TECAN plate reader. Percent viability was normalized to the vehicle control (100%).

### Robotic Screen Using CD63 Turbo-Luciferase-CD9EmGFP Reporter Cells

A total of 2210 compounds were purchased from the Maybridge Library series, including the Maybridge HitFinder library (1035 compounds) and the Maybridge HitCreator library (1175 compounds). A medium-throughput screen was performed by the SelectScreen™ service, Thermo Fisher Scientific (Madison, WI Biosciences). Briefly, the CD63Tluc-CD9EmGFP reporter cells were cultured in growth media containing 20% FBS. The cells were harvested and resuspended in assay media containing 10% Exosome-Depleted FBS (Thermo Fisher Scientific) at 2 × 10^5^ cells/ml, 50 µl/well of 384-well plates (3674, Corning). PMA (10 ng/ml at the final concentration) was used for a full stimulation (100%). Both media and vehicle (0.1% DMSO) conditions were included as negative controls. PMA and compounds (10 μM at the final concentration) were added to the cells using Echo Acoustic Liquid Handlers (Labcyte Inc. Indianapolis IN, United States), and the cells were incubated for 48 h at 37°C. Subsequently, the plate was centrifuged at 2,000 *g* for 10 min, and 25 µl supernatant was transferred to the plate (3570, Corning). TurboLuc reagent (25 µl) was added and incubated for 10 min at RT. Chemiluminescent activities were measured by the BMG CLARIOstar. The viability of cells was assessed using Presto Blue reagent (Thermo Fisher Scientific). Briefly, Presto Blue reagent (2.5 µl) was added to the cells left in the wells, and the plates were incubated for 30 min at RT. Fluorescent signals were read using the BMG CLARIOstar. The response ratio was calculated using the following formula; % response = 100 × (compound RLU-vehicle RLU)/(PMA RLU-vehicle RLU). Zʹ factor was calculated as previously reported (Zhang et al., 1999). Hit compounds were selected by a “Top X” approach in which the means of the negative control + 3SD of the unstimulated wells were calculated within each plate, and any compounds that gave signals above the cut-off were called hit compounds. Forty hit compounds were selected from the original library and used for validation studies at the UCSD laboratory.

### Statistical Analysis

Data are presented as means with a standard error of the mean (SEM) or standard deviation (SD). A two-tailed Student’s *t*-test was used to compare two groups, and one-way ANOVA with Dunnett’s post hoc was used to compare multiple groups against a control group. Spearman’s rank correlation test was performed to assess linear relationships. Analyses were performed using Prism 8 software (GraphPad Software, San Diego, CA, United States). *P* < 0.05 was considered statistically significant.

## Results

### Generation of Stable THP-1 Cells Expressing CD63-Turbo-Luciferase and CD9-EmGFP

To quantitate EV release, a human monocytic cell line with APC-like features, THP-1, was employed to generate a reporter cell line. The schematic of the process is shown in Supplementary Figure S2. A gene containing CD63 fused to Tluc, and CD9 fused to EmGFP was synthesized and cloned under the cytomegalovirus (CMV) promoter of the lentiviral vector pLenti6.3_V5-DEST_A244 (Supplementary Figures S1A,B). The transducing titer for the lenti-CD63Tluc-CD9EmGFP was ∼5 × 10^6^ transducing units/ml as determined by Lenti-X™ GoStix™. THP-1 cells were transduced with the lenti-CD63Tluc-CD9EmGFP, followed by centrifugation to improve the transduction efficiency. The transduced cells were cultured for 21 days with 5 μg/ml blasticidin. The EmGFP and Tluc expressions in the cell pool were then assessed by flow cytometer and the TurboLuc™ Luciferase One-Step Glow assay, respectively, and compared to the parental THP-1 cells. Ten percent of the antibiotic-resistant pooled cells expressed EmGFP (Supplementary Figures S1C,D), and significantly higher Tluc activity in the pooled cells was also observed (Supplementary Figure S1E). These results indicated that the antibiotic-resistant pooled cells were enriched with CD63Tluc and CD9EmGFP expressing cells.

### Cloning of Stable THP-1 Cells Expressing CD63Tluc and CD9EmGFP

**FIGURE 1:**
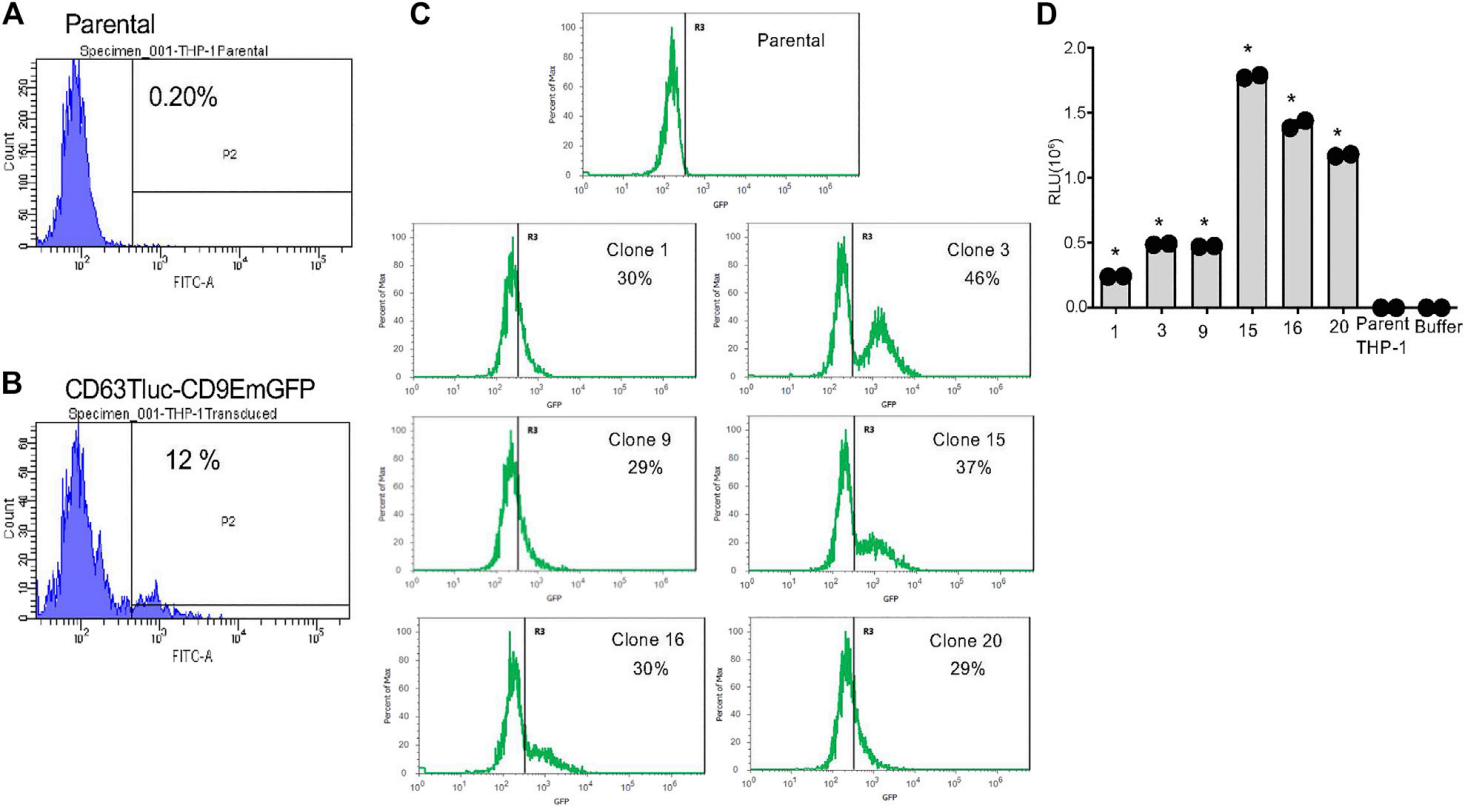
Selection of stable THP-1 transduced clones expressing CD63Tluc-CD9EmGFP. **(A,B)** EmGFP fluorescence levels of the parental THP-1 cells **(A)** and the CD63Tluc-CD9EmGFP transduced THP-1 cells **(B)** were evaluated by flow cytometric assay. The CD63Tluc-CD9EmGFP transduced THP-1 cells showed ∼12% of cells expressing high levels of EmGFP. The cells with the highest EmGFP fluorescence intensity were sorted as single cell into 96-well plates. **(C)** GFP fluorescence levels for six THP-1 clones at passage 0. GFP intensity was normalized to the maximum signal obtained in each experiment. The R3 gate represents the percentage of GFP positive cells. **(D)** Luciferase activity in selected clones at passage 4. The cells were seeded at 2.5 × 10^5^ cells/ml in a well (200 µl) of a 96-well plate and incubated overnight. Tluc activities in the culture supernatant were determined by luciferase assay and compared to the parental cells. The assays were performed in duplicate, and the data shown are means ± SD. * denotes *p <* 0.0001 by one-way ANOVA compared to the parental THP-1 cells with Dunnett’s post hoc testing.

The antibiotic-resistant pooled cells were further expanded in a medium containing 5 μg/ml blasticidin for 21 days and sorted by flow cytometry for THP-1 cell clones expressing CD9EmGFP. Approximately 12% of the pool expressed high levels of CD9EmGFP (Figures 1A,B); this subpopulation was sorted into six 96-well plates of clones, twenty-two of which were chosen for assessment of Tluc activity. Six of these clones (1, 3, 9, 15, 16 and 20) showed high expression of EmGFP (Figure 1C) and were selected for further expansion. In some clones, e.g., clone 3 and 15, the distinct EmGFP positive and negative populations were detected (Figure 1C), suggesting the presence of multiple clones. At passage 4, the EmGFP expression of the selected 6 clones dimmed markedly compared to passage 0 (Figure 1C and Supplementary Figure S3); however, the clones maintained high Tluc activity (Figure 1D). On the basis of Tluc and EmGFP activity at passage 10, clone 15 was selected for the subsequent feasibility studies of a reporter-based screening method for EV release (Supplementary Figure S4).

### Feasibility and Robustness of the Selected CD63Tluc-CD9EmGFP Transduced THP-1 Cell Clone for Compound Screening

**FIGURE 2:**
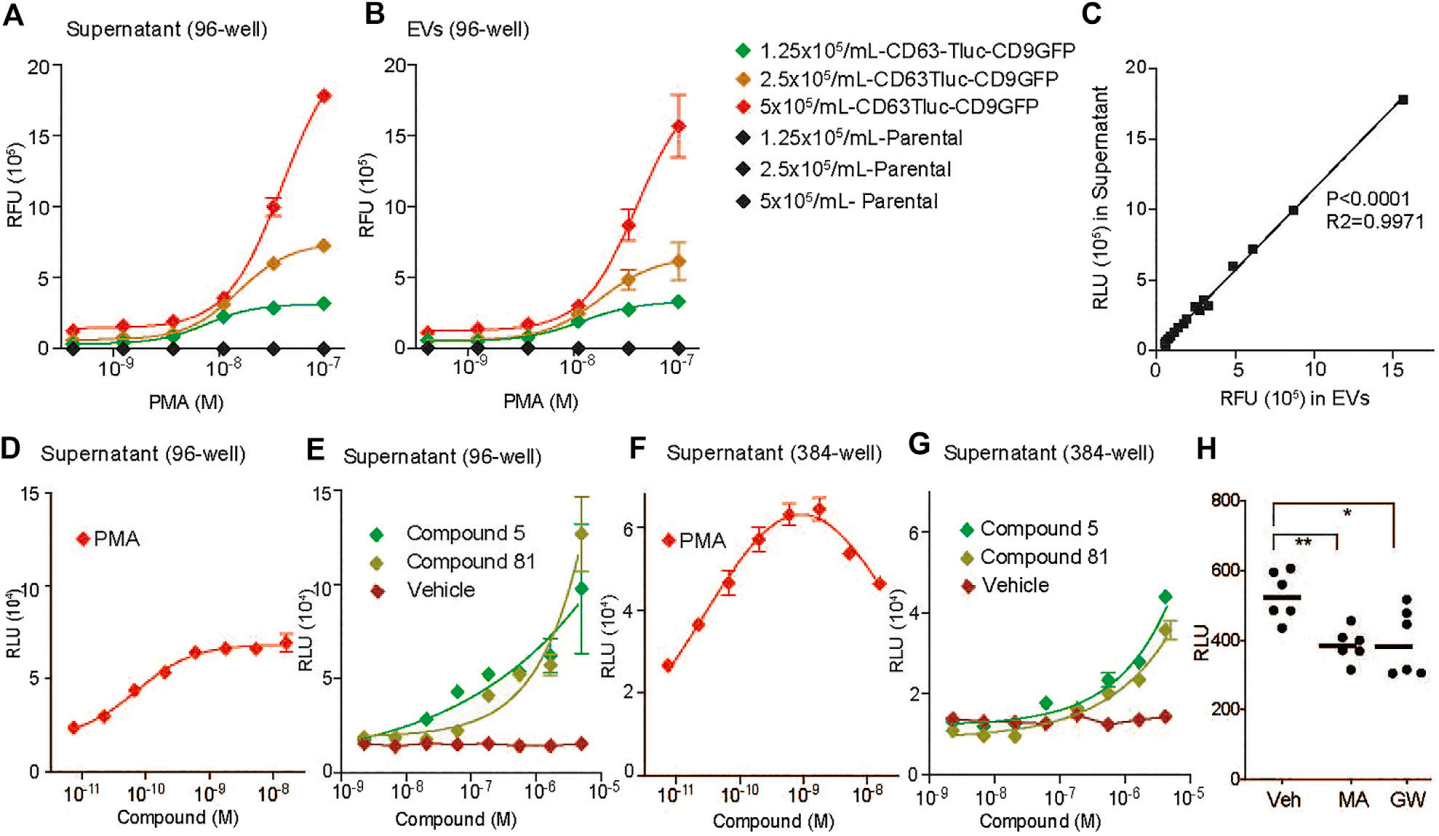
Evaluation of CD63Tluc-CD9EmGFP reporter cell clones for optimal cell number and plate formats. **(A**–**C)** Cells were seeded in duplicate at 1.25, 2.5, or 5 × 10^5^ cells/ml in 96 well-plates (200 µl/well) and stimulated with varying concentrations of PMA (3-fold serial dilution with a starting concentration of 100 nM) for 48 h. **(A)** Fifty µl of the supernatant from a total of 200 µl sample was used to detect luciferase activity. **(B)** EVs were isolated from the remaining 150 µl of each sample by total exosome isolation reagent and resuspended in 100 µl PBS. Fifty microlitre was used to measure luciferase activity. **(C)** Correlation of luciferase activities of culture supernatant and isolated EVs. *p* < 0.0001, *R* ^2^ = 0.9971 by Spearman’s correlation. **(D,E)** Luciferase activities in cell culture supernatants in 96-well format. Cells were seeded in replicates of six at 2.5 × 10^5^ cells/ml in a well of 96-well plates (200 µl/well) and stimulated with serially diluted compounds (3-fold dilutions) for 48 h. The starting concentrations of PMA was 100 ng/ml **(D)**. The starting concentration of compounds 5 and 81 was 5 µM **(E)**. Fifty microlitre of the supernatant from a total of 200 µl sample was used to measure luciferase activity. **(F,G)** Luciferase activities in cell culture supernatants in 384-well format. Cells were seeded in replicates of six at a cell density of 3.125 × 10^5^ cells/ml in a well of a 384-well plate (80 µl) and stimulated with varying concentrations of the compounds (3-fold dilutions) for 48 h. The reporter cells were stimulated with PMA **(F)**, 5, or 81 **(G)**. The starting concentration of compounds is the same as above. Fifty microlitre of the supernatant from a total of 80 µl sample was used to measure luciferase activity. Data shown are means ± SD of the assays performed in duplicates. **(H)** The CD63Tluc-CD9EmGFP reporter cells were incubated with 5 µM GW4869 (GW) or 1 µM manumycin A (MA) for 48 h. The experiments were performed in triplicates. Data shown were mean ± SEM of combined data from two independent experiments. Tluc activity in 50 µl culture supernatant was determined. * and ** denotes *p <* 0.05 and *p* < 0.01, respectively, by one-way ANOVA compared to vehicle (0.5% DMSO) with Dunnett’s post hoc testing.

To evaluate whether the CD63Tluc-CD9EmGFP transduced THP-1 cell line (clone 15, designated as the CD63Tluc-CD9EmGFP reporter cells) was sufficiently robust for a quantitative compound screening of EV release, we tested 1) the optimal cell density, 2) optimal plate format feasible for HTS and 3) DMSO tolerance. To determine an optimal cell density, we seeded CD63Tluc-CD9EmGFP reporter cells in a culture medium containing exosome depleted FBS in 96-well plates at 1.25, 2.5, or 5 × 10^5^ cells/ml and treated them with graded concentrations of PMA for 48 h. Plates were then spun at 2,000 *g* for 30 min to remove cells and debris. These supernatants were either used directly in the luciferase assay, or EVs were first isolated by the Total Exosome Isolation kit and resuspended in particle-free PBS. Samples derived from wells seeded with 5 × 10^5^/ml exhibited the best resolution and dynamic range (Figures 2A,B). Tluc activity increased in a dose-dependent manner in both culture supernatants (Figure 2A) and enriched EVs (Figure 2B). The Tluc activities in the culture supernatant and enriched EVs were positively correlated (*R* ^2^ = 0.9971; Figure 2C). To evaluate whether the CD63Tluc-CD9EmGFP reporter cells would be amenable for HTS, the assay was carried out in both 96-well and 384-well formats. The reporter cells were plated at densities of 5 × 10^5^ cells/ml in a well (200 µl) of 96-well plate or 3.125 × 10^5^ cells/ml in a well (80 µl) of a 384-well plate and were treated with serially diluted PMA and two immunostimulatory compounds (Figures 2D–G). Compounds 5 and 81 are hit compounds from a prior HTS, in which a library comprised of approximately 170,000 compounds (Small Molecule Discovery Center, University of California, San Francisco, CA, United States) was screened using THP-1 NF-κB-*bla* and THP-1 interferon-sensitive response element (ISRE)-bla reporter cell lines (Sato-Kaneko et al., 2018; Shukla et al., 2018; Chan et al., 2019). Compounds 5, and 81 were identified as prolonging NF-κB- and ISRE activation induced by LPS and type I IFN, respectively, (Sato-Kaneko et al., 2018; Shukla et al., 2018; Chan et al., 2019). PMA stimulated a dose-dependent release of Tluc activity into culture supernatants (Figures 2D,F). Compounds 5 and 81 induced EV release at concentrations above 0.55 µM (Figures 2E,G) in a dose-responsive manner. During these assays, the cells tolerated up to 0.2% dimethyl sulfoxide (DMSO). The average Zʹ value for Tluc in the supernatants of PMA and vehicle treated cells in the 384-well format was 0.63. The CD63Tluc-CD9EmGFP reporter cells were also tested for known EV release inhibitors. The reporter cells were incubated with 5 µM GW4869 (GW) or 1 µM manumycin A (MA) for 48 h and Tlu activities in the culture supernatant were examined (Essandoh et al., 2015; Datta et al., 2017; Cai et al., 2018; Datta et al., 2018). Both inhibitors significantly suppressed Tluc levels in the culture supernatant (Figure 2H). These results support the conclusion that the selected CD63Tluc-CD9EmGFP reporter cell line is amenable for screening assays using both 96-well and 384-well formats.

### Correlation of Extracellular Vesicles Release and Tluc Activities in the Culture Supernatant of CD63Tluc-CD9EmGFP Reporter Cells

**FIGURE 3:**
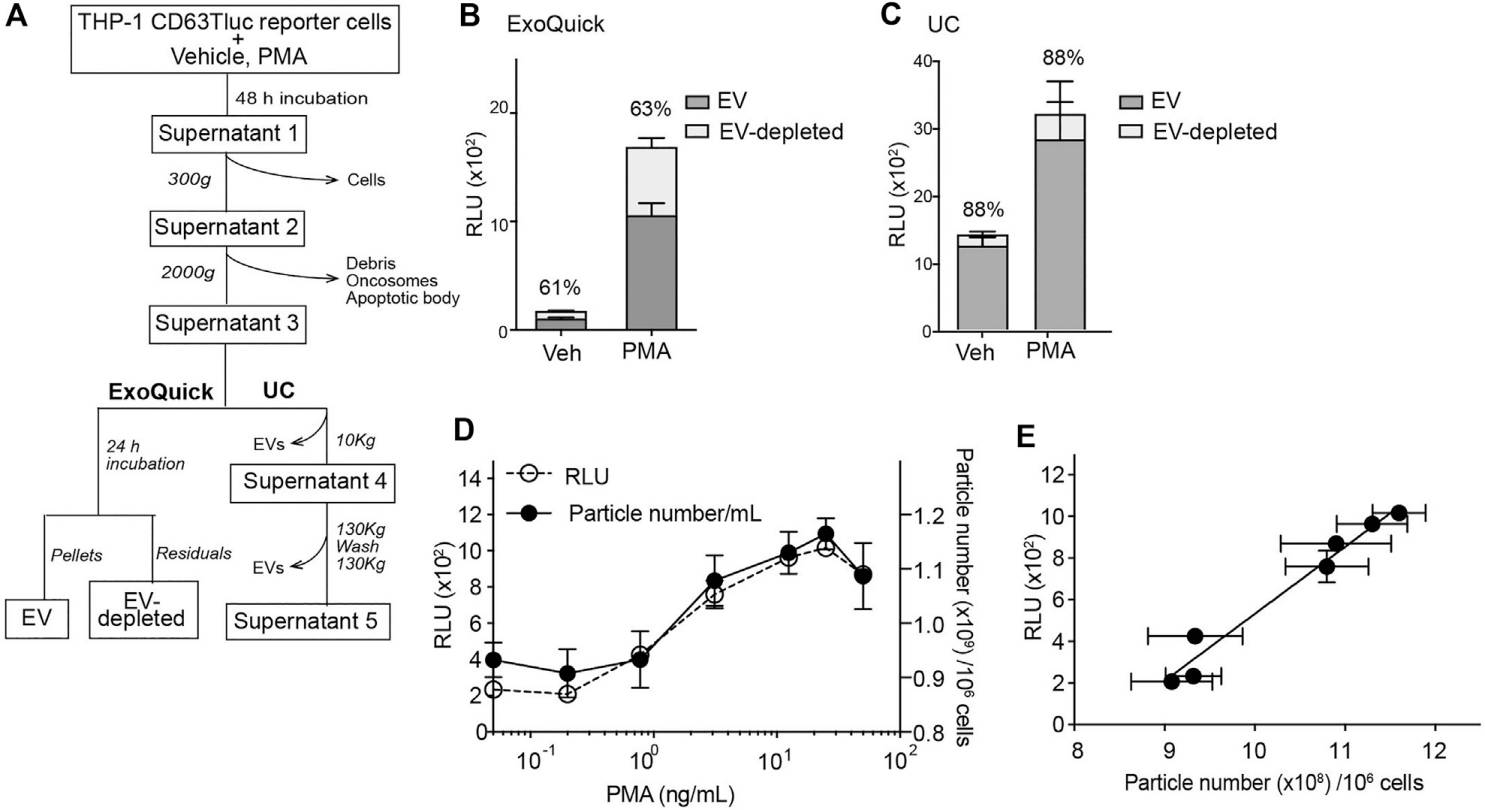
Characterization of EVs released from CD63Tluc-CD9EmGFP reporter cells. **(A)** Flowchart for the analysis of Tluc activity associated with EVs. THP-1 CD63Tluc-CD9EmGFP reporter cells (0.25 × 10^6^/ml) were treated with vehicle or PMA (50 ng/ml) for 48 h. Culture supernatants were subsequently spun at 300 g and then 2,000 g to remove cells, debris, and large vesicular bodies. TEVs were isolated from the resultant supernatants by ExoQuick-TC or ultracentrifugation (UC). **(B)** Comparison of Tluc activities in EV and EV-depleted fractions by ExoQuick-TC. Resuspended pellets were considered the EV fractions, while the residual pellet supernatants were considered the EV-depleted fractions. **(C)** Comparison of Tluc activities in EV and EV-depleted fractions by UC. Further high-speed centrifugation steps were performed to remove EVs and generate an EV-depleted solution (Supernatant 5). EV-attributable Tluc activity was indirectly determined by subtracting the Tluc activity in Supernatant 5 from Supernatant 3. Percentages represent EV-attributable Tluc as compared to total Tluc activity. **(D)** Particle numbers and Tluc activities superimposed for PMA dose-response study. CD63Tluc-CD9EmGFP reporter cells were treated with graded concentrations of PMA. EVs were isolated by ExoQuick-TC. **(E)** Correlation plot for the dependent variables assessed in Figure 4D. Particle number and Tluc activity were positively correlated (Spearman’s correlation *R* ^2^ = 0.9337; *P* < 0.0001). Data shown are means ± SEM and represent two or three independent experiments performed in duplicate or triplicate.

The above data indicated that CD63Tluc-CD9EmGFP reporter cells released EVs carrying CD63Tluc. However, because a portion of CD63Tluc may not be associated with EVs, we examined Tluc activities in the cultured cells (Table 1 and Supplementary Table S1), the EV enriched fractions (EV; Figure 3A), and the remaining supernatants (EV-depleted; Figure 3A). The CD63TLuc-CD9EmGFP reporter cells were incubated with PMA [50 ng/ml (8.17 nM)] for 48 h, and Tluc activities of the cell pellets and culture supernatants were measured (Table 1). The Tluc activities were primarily retained in the cell pellets, and only 2–4% was released into the culture supernatants (Table 1). To further test whether Tluc activities were associated with the EV fraction, the Tluc activities of isolated EVs and the residual culture supernatants were determined using two different isolation methods; ExoQuick and differential ultracentrifugation (UC) (Figures 3A–C). The percent of total Tluc activity in the EV-associated fractions was comparable across both methods (vehicle vs. PMA; 61 vs. 63% by ExoQuick; 88 vs. 88% by UC) (Figures 3B,C). Furthermore, a dose-response study using graded concentrations of PMA showed that the luciferase activities and particle numbers in the culture supernatants were positively correlated (Spearman’s correlation *r* = 0.9339, *p* < 0.0001, Figures 3D,E).

### Characterization of Extracellular Vesicles Released by CD63 Tluc-CD9EmGFP Reporter Cells

**FIGURE 4:**
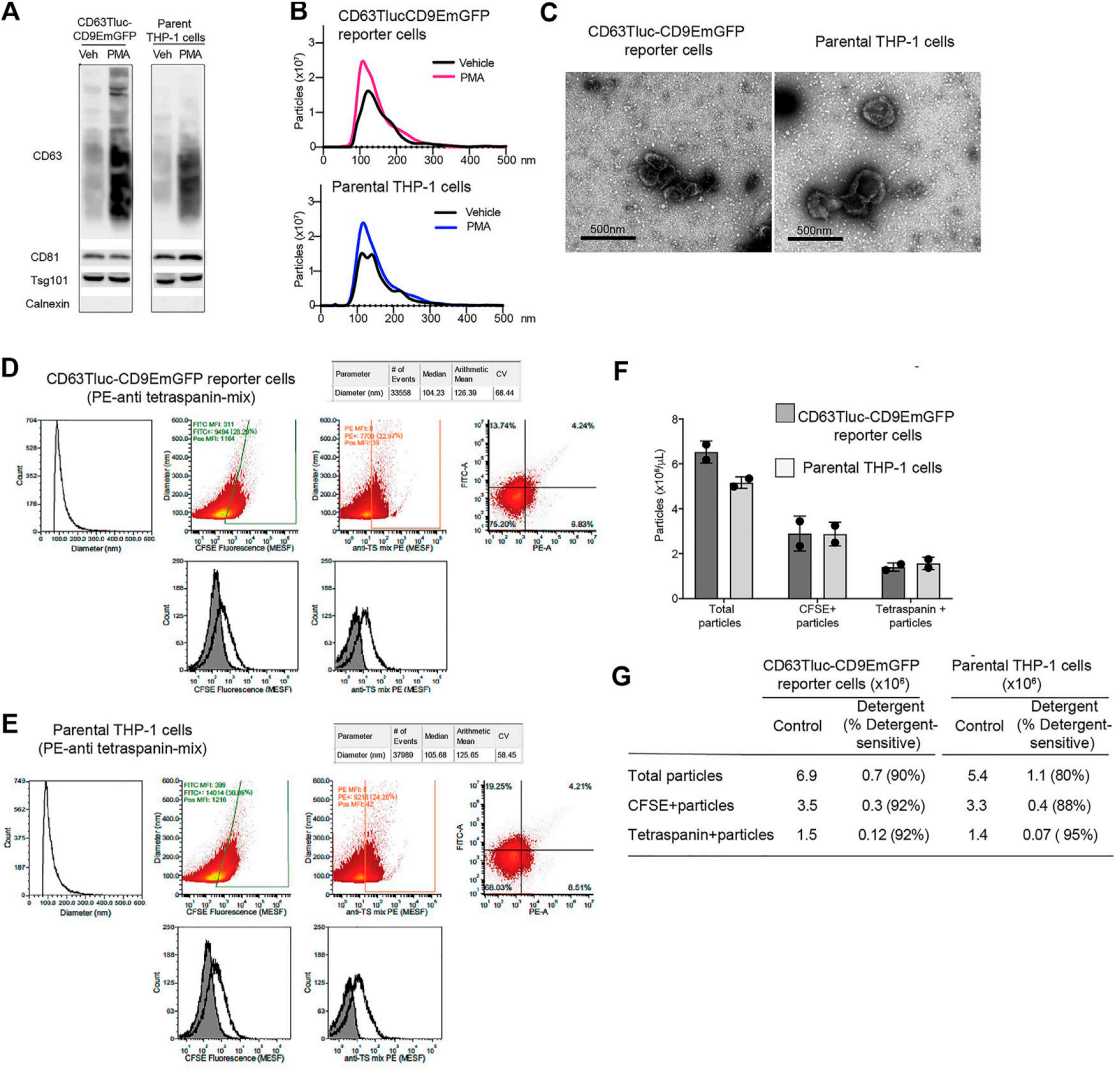
Comparison of EVs released from CD63Tluc-CD9EmGFP reporter cells and parental THP-1 cells. **(A–C)** 0.5 × 10^6^ cells/ml CD63Tluc-CD9EmGFP reporter cells or parental THP-1 cells were treated with 50 ng/ml PMA, 5 µM compounds 81, or 5 for 48 h. EVs were isolated using ExoQuick and were resuspended in particle-free PBS to 1/500 of the original volume of culture supernatant. **(A)** Immunoblot of EVs isolated from CD63Tluc-CD9EmGFP reporter cells and parental THP-1 cells. EVs (30 µg protein/well) were separated in a 4–12% NuPAGE gel. Blots were probed with anti-CD63, anti-CD81, anti-Tsg101, or anti-Calnexin Abs. The images shown are representative of three independent experiments. The original blots are presented in Supplementary Figure S4. **(B)** Size distributions of isolated EVs released from CD63Tluc-CD9EmGFP reporter cells **(top)** and parental THP-1 cells following treatment with PMA (50 ng/ml) or vehicle as measured by NanoSight. **(C)** Morphological examination of EVs from vehicle-treated CD63Tluc-CD9EmGFP reporter cells and parental THP-1 cells by transmission electron microscopy. Scale bars represent 500 nm. **(D–G)** Vesicle flow cytometric (vFC) analysis of EVs isolated from CD63Tluc-CD9EmGFP reporter cells **(D,F,G)** or parental THP-1 cells **(E–G)** using differential ultracentrifugation. EVs were stained with vFRed™, CFSE, or a mixture of anti-TS antibodies. The gating strategy, positive and negative control samples and isotype staining are presented in Supplementary Figure S8. **(F)** The number of total EVs are reported (vFRed™-positive), as are the number of CFSE-positive and TS-positive EVs that stained above an arbitrarily placed threshold determined from the background of the relevant unstained samples. **(G)** The vesicular nature of the particles was validated by detergent treatment (0.1% Empigen, Supplementary Figure S9). **(A**–**E,G)** The data shown are representative of two independent experiments. **(F)** Means ± SD of two independent experiments are presented.

As the engineered CD63Tluc-CD9EmGFP reporter cells constitutively express CD63 driven by a CMV promoter, we examined whether overexpression of CD63 attenuated EV biogenesis and release. We compared the quantity and phenotype of EVs released from the CD63Tluc-CD9EmGFP reporter cells and parental THP-1 cells using four approaches; immunoblotting, NTA, TEM, and vesicle flow cytometry. The expression of TSs (CD63, CD81, and Tsg101) was evaluated by immunoblotting (Figure 4A and Supplementary Figure S5 for the original blots). The EV expression of CD63, CD81, and Tsg101 were similar in the CD63Tluc-CD9EmGFP reporter cells and parental THP-1 cells (Figure 4A). Calnexin, an endoplasmic reticulum-associated protein, was not detected in any EV samples (Figure 4A). Particle concentrations and mean diameters of isolated EVs were determined by NanoSight (Table 2). The mean sizes of isolated EVs from the vehicle- or PMA-treated CD63Tluc-CD9EmGFP reporter cells were comparable to EVs released by the parental THP-1 cells (Table 2). The size distribution of EVs was similar between the reporter cells and parental THP-1 cells (Figure 4B). Isolated EVs from parental THP-1 cells and CD63Tluc-CD9EmGFP reporter cells were also morphologically similar as evaluated by TEM and predominantly had diameters within the size distributions detected by NTA (Figure 4C and Supplementary Figure S6).

EVs released by CD63Tluc-CD9EmGFP reporter cells and parental THP-1 cells were further characterized by vesicle flow cytometry. The EVs were detected by their membrane staining vFRed™ fluorescence, as well as their intra-vesicular esterase activity using CFSE and their expression of common TS using a mixture of PE-conjugated anti-TS (CD9, CD63, and CD81) antibodies. Essential controls performed to demonstrate single EV specificity include buffer and reagent-only controls, dilution series to determine dynamic assay range and lack of coincidence, and detergent lability testing (Supplementary Figures S7–S10). EVs ranged in size from ∼75 nm, a typical limit of detection for vFC on the CytoFlex, to about 250 nm (median ∼105 nm). EVs from both reporter and parental THP-1 cells contained esterase activity, as detected by CFSE (Figures 4D,E). CFSE staining resulted in single EV fluorescence intensities that ranged from less than ∼200 FITC molecule of an equivalent soluble fluorophore (MESF), the limit of detection (LOD) defined by background autofluorescence, to several thousand FITC MESF per EV (mean ∼1500 MESF, mean ∼3000 MESF), with ∼40–50% positive above an arbitrarily set threshold of ∼200 FITC MESF. As measured by immunofluorescence, EVs also bore surface TS molecules with a mix of anti-CD9, CD63, and CD81 PE-labeled antibodies. EVs from both cells stained for TS, with single EV intensities that ranged from less than 20 PE MESF, the LOD defined by background autofluorescence of unstained EVs, to ∼300 MESF per EV (median 8 MESF, mean ∼30 MESF) (Figures 4D,E). Approximately 20–25% of anti-TS PE-stained EVs were positive above an arbitrary threshold of ∼20 PE MESF. Equivalent number of EVs were released from the reporter cells and the parental THP-1 cells (Figure 4F). Over 90% of TS positive particles were detergent sensitive in both cell lines as expected for EVs (Figure 4G; Supplementary Figure S9). Taken together, EVs released from CD63Tluc-CD9EmGFP reporter cells and parental THP-1 cells were similar in size, numbers, and EV components.

### Compound Screen Using CD63Tluc-CD9EmGFP Reporter Cells

**FIGURE 5:**
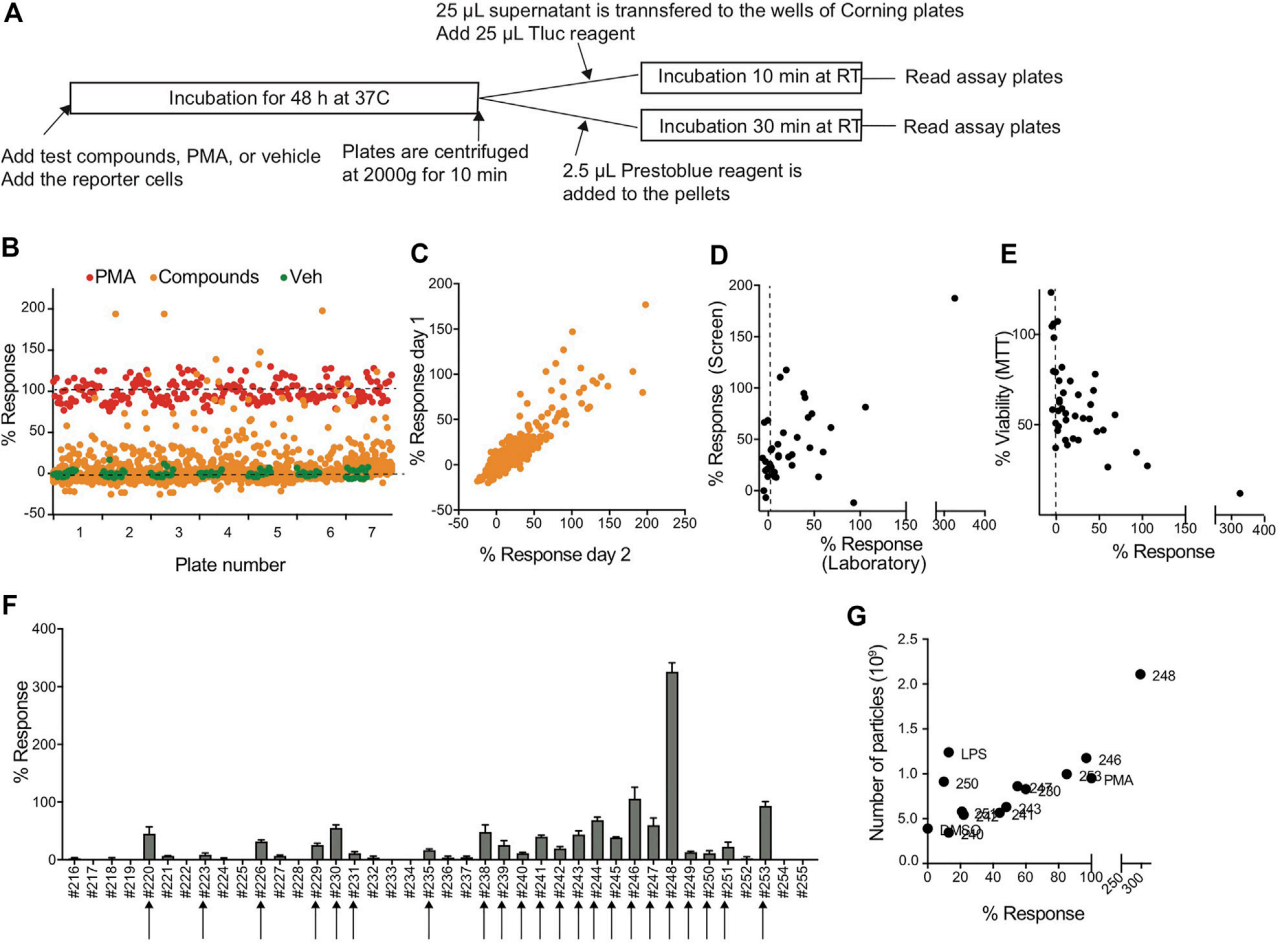
Compound screen using CD63Tluc-CD9EmGFP reporter cells. **(A)** Outline of the compound screen. CD63Tluc-CD9EmGFP reporter cells were incubated with test compounds. PMA (10 ng/ml at the final concentration) was used for a full stimulation (100%). Both media and vehicle (1% DMSO) were included in negative controls. The plates were incubated at 37°C for 48 h, and the Tluc activities in the culture supernatant were measured. 2.5 µl Presto Blue reagent was added to the cells left in the wells, and the plates were incubated for 30 min at RT. The screens were performed on days 1 and 2. **(B)** Example of the Tluc activity distribution of the screen (day 1). Orange circle; test compounds, red circle; PMA, and green circle; vehicle control. The response ratio was calculated using the following formula; % response = 100 × (compound RLU-vehicle RLU)/(PMA RLU-vehicle RLU). **(C)** Reproducibility of % response of day 1 vs. day 2 (Spearman’s correlation, *r* = 0.4912, *P* < 0.0001). **(D)** % responses of compounds in the SelectScreen and the UCSD laboratory positively correlated (Spearman’s correlation, *r* = 0.4445, *p* = 0.004). **(E)** Cytotoxicity of compounds was determined by MTT assay in the UCSD laboratory and expressed as % of control (vehicle 0.1% DMSO). The data were plotted with % response (Spearman’s correlation *r* = −0.5728, *p* = 0.0001). **(F)** Validation of the hit compounds. Representatives of selected hit compounds were re-tested in the UCSD laboratory. Twenty-two compounds out of 40 compounds were confirmed. **(G)** EVs were enriched by 8% PEG precipitation method and the numbers of particles were measured by an nCS1 Instrument. The number of particles and % response were positively correlated (Spearman’s correlation, *r* = 0.5875, *p* = 0.03).

To evaluate whether the CD63Tluc-CD9EmGFP reporter cells were acceptable for HTS, we transferred the reporter cells to the Thermo Fisher SelectScreen Facility (Madison, WI), where the protocols were further optimized for HTS using their robotic instruments (Figure 5A). A total of 2210 compounds on seven plates were screened twice on separate days. The scatter plots showed that the signal window was adequate between the positive control (PMA) and negative control (0.1% DMSO), and the average Z′ factor was 0.54 ± 0.07 (mean ± SEM) (Figure 5B and Supplementary Figure S11A,B). The confirmation rate of the two assay dates (days 1 and 2) was 78.6%, and % response of day 1 and 2 data points were positively correlated (Spearman’s correlation *r* = 0.4912, *p* < 0.0001 two-tailed) (Figure 5C). The hit rates of the day 1 and 2 screens were 10 and 11%, respectively (Table 3). One hundred eighty-seven compounds that showed % response above the cut-off value (mean of negative control values + 3SD) in days 1 and 2 were selected as hit candidates.

**TABLE 3:** Number of hit, and non-hit compounds.

		Day 1	
		Non-hit	Hit
Day 2	Non-hit	1909	63
	Hit	51	187

Forty out of the 187 compounds were selected from the original library plates and re-tested to induce Tluc activity in the same reporter cell line in the UCSD laboratory. The percent response ratios measured at the SelectScreen facility and our manual rescreen positively correlated (Spearman’s correlation *r* = 0.4445, *p* = 0.004 two-tailed (Figure 5D). A negative correlation between cell viability and % response was observed in the validation study (Spearman’s correlation *r* = −0.5728, *p* = 0.0001, Figure 5E). Twenty-two of the 40 re-tested compounds had confirmed activity using the same cut-off values (means of negative controls + 3SD (arrows in Figure 5F). We further confirmed for representative compounds that the % response positively correlated to particle numbers (Spearman’s correlation *r* = 0.5875, *p* = 0.03, two-tailed, Figure 5G) as determined by nCS1 Instruments (Spectradyne). When we compared % cell viability of top five compounds, compound #244, which presented with more than 50% cell viability after 48 h incubation, was chosen as a possible hit compound (Supplementary Figure S11C). Collectively, the CD63Tluc-CD9EmGFP reporter cells were amenable to a robotic HTS operation and provided wide signal windows between positive and negative controls, excellent Z′ factors, and a positive correlation of EV release and % response, indicating that this reporter cell line is suitable for large screens.

## Discussion

EVs are lipid-bilayer-bound vesicles that have multiple biological functions in mediating cell to cell communications (Margolis and Sadovsky, 2019). Recently the role of EVs in initiating and regulating immune responses has generated interest in immunotherapy. EVs from APCs or diseased cells can present antigens to T and B cells or enhance the functions of immune cells as a by-stander effect (Segura et al., 2005; Luketic et al., 2007; Wen et al., 2017). Hence, pharmacological agents that reliably influence the biogenesis, biophysical properties, and physiological functions of EVs could add to immune-based therapeutic strategies. In this study, we aimed to develop a cell-based screening system for EV release from immune cells. We generated a luciferase reporter linked to CD63 (CD63Tluc) in a human monocytic cell line, THP-1. The cloned CD63Tluc-CD9EmGFP reporter cells, when treated with a known stimulus, shed EVs at a level comparable to that of the parent cell line and with similar phenotypic and morphological properties. The number of EVs measured by NTA correlated with the Tluc activity in culture supernatants. Also, Tluc activity in the culture supernatant of reporter cells increased in a dose-dependent manner following stimulation with either PMA or two other immunostimulatory compounds.

In the design of the reporter construct, we co-opted the well-described essential roles of TSs in EV biogenesis (Petersen et al., 2011; van Niel et al., 2011; Andreu and Yanez-Mo, 2014; Datta et al., 2018). CD63 is a member of the TS family of proteins transferred from the plasma membrane to endosomal compartments and also delivers selected cargo to intraluminal vesicles for exocytosis (Stoorvogel et al., 2002; Pols and Klumperman, 2009). The reporter construct contains the CD63 fusion gene downstream from the CMV promotor, resulting in a slight increase in CD63 protein expression (Figure 4A). Other groups have used CD63 for reporter constructs for EV research (Datta et al., 2018; Cashikar and Hanson, 2019). Cashikae et al. developed a plasmid-based construct with luciferase that delivered an EV-associated signal in several cell types indicating that the choice of CD63 is sufficiently robust for cell-based reporter constructs (Cashikar and Hanson, 2019). Datta et al. optimized the CD63 reporter cells in a prostate cancer cell line C4-2B that measure cellular CD63 biogenesis in a HTS format (Datta et al., 2018). Our data, however, showed that CD63Tluc-CD9EmGFP THP-1 cells were sensitive enough to quantitatively measure CD63-carrying EVs directly in culture supernatant.

We initially attempted to prepare dual reporter cells using the CD63Tluc-CD9EmGFP construct. However, GFP fluorescence by the transduced THP-1 cells was dimmed after 4th passage and did not recover thereafter. The CD9EmGFP fusion may have been driven off of the IRES element, and there may have been incomplete transcription after repeated passages and cell division. Alternatively, overexpression of GFP, particularly with an SV40 promoter, might have impaired cell survival (Liu et al., 1999). Although overexpression of EmGFP or CD9 was not reported as toxic in THP-1 cells, it is fully surmisable that the overexpression of the fused protein attributed to the cytotoxicity of THP-1 cells. To test EV induction we used PMA, a protein kinase C activator, which increases intracellular Ca^2+^ levels in THP-1 and other cell types (Reisine and Guild, 1987; Gonczi et al., 2002; Yu et al., 2003). PMA stimulates increased expression levels of CD63 compared to vehicle-treated THP-1 cells (Chakraborty et al., 2019) and the increase in intracellular Ca^2+^ generates exosome secretion (Savina et al., 2003; Messenger et al., 2018). These reports are consistent with our finding that PMA enhanced CD63 expression in both parent THP-1 and the reporter cells and led to the choice of PMA as a positive control for the HTS.

To characterize EVs from the reporter line, we used four enrichment methods: Total Exosome Isolation Reagent™, ExoQuick, 8% PEG precipitation, and differential ultracentrifugation. The differential ultracentrifugation has been recognized as the “gold standard” purification method consisting of low-speed centrifugation to remove live and dead cells and cell debris and high-speed ultracentrifugation to pellet EVs. Total Exosome Isolation Reagent, ExoQuick, and 8% PEG precipitation method use a principle of PEG precipitation that co-precipitate proteins and nucleic acids in the environment (Garcia-Romero et al., 2019). Tluc activities were more highly retained in the EV fractions of differential ultracentrifugation samples, compared to ExoQuick samples. This finding suggests that the isolation methods could significantly contribute to the apparent phenotypes of isolated EVs (Bozic et al., 2019; Garcia-Romero et al., 2019; Brennan et al., 2020).

Further characterization by immunoblot and single vesicle flow cytometry demonstrated that TSs were expressed on the surface of individual EVs. The number, size distribution, and TS staining were similar for EVs released from the reporter cells and parental THP-1 cells, as was staining with CFSE, a marker for cytoplasmic esterases inside the EVs. EV anti-TS PE fluorescence ranged from less than 20 MESF, the LOD defined by background autofluorescence of unstained EVs, to ∼300 MESF per EV, which estimated the number of antigen epitopes to within a factor of two, given the bivalent nature of the IgGs used. Approximately 20–25% percent of EVs were positive above an arbitrary threshold of ∼20 MESF, but the characterization of EVs as “positive” or “negative” in this context is inappropriate, owing to the vagaries of making measurements near the detection limit of the instrument. CFSE staining, which is often considered a “generic” marker of EVs, ranged from less than ∼200 FITC MESF, the LOD defined by background autofluorescence, to several thousand FITC MESF per EV, with ∼40–50% positive above an arbitrarily set threshold of ∼200 FITC MESF. As for immunofluorescence measurements of EVs, it is inappropriate to refer to these as CFSE “positive” and “negative” EVs, as these values depend upon the performance features of the instrument as much as the properties of the EVs and we use them here simply as a marker of EVs with entrapped cytoplasm.

Our goal for developing robust reporter cells measuring EV release from immune cells is to screen for compounds that enhance or suppress EV release into tissue culture supernatant. We assessed the robustness of the responses after the prolonged culture. The luciferase activities remained stable in the CD63Tluc-CD9EmGFP reporter cells for over 30 passages. The cell numbers and conditions were miniaturized to a 384-well format suitable for a robotic HTS operation (Datta et al., 2018; Im et al., 2019). One of the limitations of a HTS study is the small number of replicates; however further confirmation in subsequent studies with additional assays were used here to support the key findings. The screening data of 2210 compounds demonstrated that the assays using CD63Tluc-CD9EmGFP reporter cells were reproducible and showed a wide assay window and excellent Z′ factors. Compound #244, 3-(3- (methylthio)-1-phenyl-1H-pyrazol-4-yl)-5-(thiophen-2-yl)-1,2,4-oxadiazole, was chosen as a possible hit candidate and carried out for the validation study. This compound had no prior reported biologic activity; however, 1,2,4-oxadiazole derivatives are reported to show a broad range of biologic activities, e.g., anti-tumor, anti-microbial, and anti-inflammatory, anti-allodynic, anti-convulsant, anti-Alzheimer agents, anti-insomnia, and others (Biernacki et al., 2020).

The validation study using 184 confirmed hit candidates showed that the cytotoxicity data and % response ratio were negatively correlated, suggesting cytotoxic compounds enhance EVs release (Figure 5E). The exosome function from dying or apoptotic cells was recently discussed (Kakarla et al., 2020). Dying cells due to infection or tissue injury, release EVs that transfer the proinflammatory information to the recipient cells (Baxter, 2020). THP-1 cells that are undergoing lytic cell death release more EVs compared with viable or apoptotic cells *in vitro*, and it is essential to distinguish between Tluc associated with EV and free Tluc (Baxter et al., 2019). Therefore, it is likely that the cytotoxicity of the compounds causes higher EV release. It is also essential to distinguish Tluc associated with EVs and free Tluc. Our study indicated that only 60–88% Tluc was associated with EV fractions in the reporter cell culture supernatants following vehicle or PMA stimulation. After the treatment with cytotoxic compounds, the proportion of free Tluc in the total Tluc in the culture supernatants might be attenuated by incubation time and compound concentration. Hence, in the HTS, further validation screening of confirmed hit candidates using NTA or nanoparticle flow cytometry is indispensable for properly identifying the hit compounds.

In summary, we developed a human reporter cell line to quantitatively measure EV release from APCs. A CD63Tluc-CD9EmGFP reporter construct was transduced into the human monocytic cell line, THP-1. The Tluc activities in the culture supernatant positively correlated with the number of EVs released by the reporter cells as measured by NTA. Tluc assay using the CD63Tluc-CD9EmGFP reporter cells was sensitive and amenable to a 384-well robotic screening format with a Z′ factor over 0.5. These results indicated that the CD63Tluc-CD9EmGFP reporter cell line exhibited reliable, reproducible, and robust responses that makes it an excellent tool for the screening of compounds that enhance EV release by APCs.

## Data Availability

The original contributions presented in the study are included in the article/Supplementary Material, further inquiries can be directed to the corresponding author.
