# Supplementary material for: Generation and Application of a Reporter Cell Line for the Quantitative Screen of Extracellular Vesicle Release
Source: Front Pharmacol. 2021 Apr 16;12:668609. doi: 10.3389/fphar.2021.668609 (PMC8085554; doi:10.3389/fphar.2021.668609)
Supplement: Supplementary file 1 [file datasheet1.pdf]

SUPPLEMENTAL INFORMATION FOR FLOWCYTOMETRIC EV ANALYSIS

Supplemental Material and Methods

Vesicle Flow Cytometry

Single vesicle flow cytometry was performed using a commercial assay kit (vFC™ EV Analysis Kit, Cellarcus Biosciences; Supplemental Table 1) and flow cytometer (CytoFLEX S, Beckman Coulter). Sample staining was performed in 96-well v-bottom plates as directed in the vFC™ Protocol. A fresh 10× vFRed™ stain solution was prepared from the 100× stock in vesicle Staining Buffer. Staining reactions consisted of 5 µl diluted sample optimal dilution (determined in preliminary measurements), 5 µl vFRed™ 10x stain solution, and 5 µL 10× FL-marker (FL-antibody) added to a total volume of 50 µL in vesicle Staining Buffer. Samples were incubated for 1 h at ambient temperature, followed by a post-stain dilution (1000-fold) for analysis on the CytoFLEX.

Supplemental Table 1. Reagents for EV flow cytometric assays

Reagent [clone]	Source	Catalog number
vFC™ EV Analysis Assay kit	Cellarcus Biosciences	CBS4
CFDA SE, 5 and 6-isomers	Cellarcus Biosciences	CBS4P
Anti-TS PE mix	Cellarcus Biosciences	CBS5-PE-20T
Anti-CD9 [HI9a] PE	Cellarcus Biosciences	CBS10-PE-20T
Anti-CD63 [5A6] PE	Cellarcus Biosciences	CBS11-PE-20T
Anti-CD81 [Hip9] PE	Cellarcus Biosciences	CBS12-PE-20T
Anti-CD82 [ASL_24] PE	Cellarcus Biosciences	CBS14-PE-20T
Anti-CD151 [50_6] PE	Cellarcus Biosciences	CBS15-PE-20T

Flow cytometer configuration and operation. The CytoFLEX flow cytometer was configured to measure violet side scatter (VSSC) as described in the CytoFLEX Instructions for Use (https://www.beckman.com/techdocs/B49006AP/wsr-168786). Briefly, the Violet 405nm filter is placed in position 2, the Violet 450nm filter in position 3, and an unused filter in position 1. The complete detector configuration with filters and gains is presented in Supplemental Table 2. The instrument fluorescence response was calibrated in units of Molecules of Equivalent Soluble Fluorochrome (MESF) in the PE (561-585/42) and FITC channels (488-525/40) using hard-dyed calibration beads (nanoRainbowcalibration particles, Cellarcus) that had been cross-calibrated against PE

(PE QuantiBrite beads, BD Biosciences #340495) and FITC (Quantum FITC MESF beads, Bangs Labs #555) MESF standards on the same instrument. Sample was introduced at the HIGH sample flow rate (60 µL/sec, confirmed using Spherotech AccuCount beads) and data acquisition was triggered by the 488 nm-excited red fluorescence of the membrane stain, with a threshold set to accept ~2 events/second with a buffer-only sample, and ~20 events/sec for a buffer +vFRed™ sample. Each sample well was analyzed for 120 seconds.

**Supplemental Table 2. CytoFLEX S detector configuration**			
Detector	Parameter	Stain Name	Gain
V-1	405-780/60		1000
V-2	405-405/10	VSSC	100
V-3	405-450/45		1000
V-4	405-525/40		1000
V-5	405-610/20		1000
V-6	405-660/20		1000
R-1	640-660/20		1000
R-2	640-712/25		1000
R-3	640-780/60		1000
B-1	488-488/8	SSC	100
B-2	488-525/40	FITC	1000

B-3	488-690/50	vFRed	1000
Y-1	561-561/10		1000
Y-2	561-610/20		1000
Y-3	561-585/42	PE	1000
Y-4	561-690/50		1000
Y-5	561-780/60		1000

Vesicle gating and data analysis. Data were analyzed using a standardized data analysis created in FCS Express version 7 (De Novo Software). The first 20 seconds of data were discarded via a Time gate (**Supplemental Figure 7A**) due to a consistent but unexplained background event anomaly observed on several different CytoFLEX instruments. The remaining 100 seconds of data, corresponding to 100 µL of measured sample, and a plot of vFRed-A vs vFRed-H used to set a gate (**Supplemental Figure 7B**) that exclude certain background events that can be identified by their lower signal pulse area and widths. These events were further gated (Vesicle gate) to include events with membrane fluorescence and light scatter intensity characteristic of EVs, and to exclude high light scatter intensity background events that have been noted in certain samples (**Supplemental Figure 7C**). Events counts in the Vesicle gate in 100 ul analyzed were used to estimate the concentrations in plasma after accounting for the pre-stain and post-stain dilutions.

Vesicle size calibration. Vesicle size was estimated using the fluorescence from a vesicle size standard (Lipo100, Cellarcus) whose size distribution was measured by nanoparticle tracking analysis (NTA, NanoSight, Malvern) and resistive pulse spectroscopy (RPS, nCS-1, Spectradyne). The Lipo100 standard was stained and measured under the same conditions as samples, and the relationship between population surface area (estimated by NTA, **Supplemental Figure 7D**) and membrane fluorescence (measured by FC, **Supplemental Figure**

7F) was used to calculate the surface are per fluorescence intensity unit (**Supplemental Figure 7G**). This factor was used to calibrate the membrane fluorescence axis in units of surface area (**Supplemental Figure 7H**) and diameter (**Supplemental Figure 7I**), assuming spherical particles. The vesicle size detection limit, estimated from the calibrated trigger threshold surface area and diameter detection, is ~75 nm.

Controls for single vesicle analysis. The specificity of single vesicle analysis was evaluated via several control measurements. Buffer-only and buffer plus membrane stain showed low levels of background events (~200 and 2000 in ~100 seconds of gated data; **Supplemental Figure 7I**), while detergent treatment (0.1% Empigen added before the post-stain dilution) resulted in lysis of >90% of gated events (**Supplemental Figure 8D**), indicating the vast majority of detected events were detergent-labile, as expected for EVs. Serial dilution of sample showed the expected proportional decrease in detected events with minimal change in the brightness of those events (**Supplemental Figure 8**), consistent with the measurement of single EVs.

Vesicle immunofluorescence calibration, controls, and reporting. EV immunofluorescence was calibrated in MESF units for FITC and PE conjugates, as described above. Immunofluorescence negative controls included the Lipo100 vesicle standard, and an isotype control antibody demonstrated undetectable binding by Fc receptors. The median fluorescence intensity of the entire positive EV population, the number of positive EVs with fluorescent intensity higher than the upper threshold of an unstained sample (<0.5% "positive"), and the MFI of positive EVs were calculated using FCS Express version 7.

Cytometry Part A
Author Checklist: MIFlowCyt-Compliant Items

Requirement	Please Include Requested Information
1.1. Purpose	To enable drug discovery and characterization of compounds that affect EV biogenesis, function, and release, we developed and characterized a reporter cell line that allows the quantitation of EV shed into culture media.
1.2. Keywords	Extracellular vesicle, exosome, THP-1, CD63, CD9, luciferase, antigen-presenting cells
1.3. Experiment variables	NA
1.4. Organization name and address	University of California San Diego, 9500 Gilman Dr., La Jolla, CA 92093-0809
1.5. Primary contact name and email address	Tomoko Hayashi, MD Ph.D, thayashi@ucsd.edu
1.6. Date or time period of experiment	Jan 2019 – June 2020
1.7. Conclusions	These results indicate that the use of CD63Tluc-CD9EmGFP reporter cells is feasible for HTS of compounds that regulate EV release.
1.8. Quality control measures	Instrument performance was characterized using a combination of multi-intensity single fluorophore beads (Quantum FITC, Bangs Labs Quantibrite PE, BD Biosciences) whose intensity had been calibrated in units of MESF, multi-intensity multifluorophore beads (vCal nanoRainbow, Cellarcus), and antibody capture beads (vCal antibody capture beads, Cellarcus) calibrated to report results in units of antibodies bound per vesicle (ABV). EV analysis by vesicle flow cytometry (VFC) was conducted and reported as suggested by the MIFlowCyt-EV guidelines (See attached checklist).
2.1.1.1. (2.1.2.1., 2.1.3.1.) Sample description	Cell culture supernatants
2.1.1.2. Biological sample source description	Human monocytic cell line THP-1 cells and THP-1 cells transduced with CD63Tluc-CD9EmGFP reporter construct
2.1.1.3. Biological sample source organism description	Human
2.1.2.2. Environmental sample location	NA
2.3. Sample treatment description	

2.4. Fluorescence reagent(s) description	Supplemental Table 1 , Supplemental information
3.1. Instrument manufacturer	BDBiosciences FACSAria, Beckman Coulter CytoFlexS
3.2. Instrument model	FACSAria CytoFlexS
3.3. Instrument configuration and settings	The CytoFlex flow cytometer with stock filters (see table below) was configured to measure violet side scatter (VSSC) as described in the CytoFLEX Instructions for Use (https://www.beckman.com/techdocs/B49006AP/wsr-168786). Briefly, the Violet 405nm filter is placed in position 2, the Violet 450nm filter in position 3, and an unused filter in position 1. The gain on all scatter channels was set to 100, the gain on all fluorescence channels was set to 1000.<br>(Supplemental Table 2)<br>DetectorLaser-Center/width Name<br>V-1 405-780/60<br>V-2 405-405/10 VSSC<br>V-3 405-450/45<br>V-4 405-525/40<br>V-5 405-610/20<br>V-6 405-660/20<br>R-1 640-660/20<br>R-2 640-712/25<br>R-3 640-780/60<br>B-1 488-488/8 SSC<br>B-2 488-525/40 FITC<br>B-3 488-690/50 vFRed<br>Y-1 561-561/10<br>Y-2 561-610/20<br>Y-3 561-585/42 PE<br>Y-4 561-690/50<br>Y-5 561-780/60
4.1. List-mode data files	*We recommend all authors to submit their data files to http://flowrepository.org and to make them available for the peer-review process. If you have done so, please let us know by inserting the following codes (replace the red text):<br><br>1) The link for peer-review process:<br>https://flowrepository.org/id/RvFrRAZHw9PqF5U3zdTFTXCB0uWM66FTgZa451GvplEljIIu4Oj9yV5VLArFcSGC..<br>This link will only be shared with reviewers of your manuscript.
4.2. Compensation description	No compensation was performed. Single stained controls showed lack of detectable spillover.
4.3. Data transformation details	
4.4.1. Gate	Data were analyzed using FCS Express (De Novo Software). The first 20 seconds of data were

description	discarded via a Time gate (SFigure 7) due to a consistent but unexplained background event anomaly observed on several different CytoFlex instruments. The remaining 100 seconds of data, corresponding to 100 uL of measured sample, and a plot of vFRed-A vs cFRed-H used to set a gate (SFigure 7) excluding certain background events that could be identified by their lower signal pulse area and widths. These events were further gated to include events with membrane fluorescence and light scatter intensity characteristic of EVs, and to exclude high light scatter intensity background events that have been noted in certain samples (SFigure 7).
4.4.2. Gate statistics	EV immunofluorescence data was analyzed to calculate the median fluorescence intensity of the entire EV population, the number of EVs with immunofluorescence positive above gate position at the upper threshold of an unstained sample (<0.5% "positive"), and the (MFI) of these positive EVs.
4.4.3. Gate boundaries	EV immunofluorescence data was analyzed to calculate the median fluorescence intensity of the entire EV population, the number of EVs with immunofluorescence positive above gate position at the upper threshold of an unstained sample (<0.5% "positive"), and the (MFI) of these positive EVs.

Notes

Feel free to use more space than allocated.

You can embed graphics/figures in this document, if needed.

Please make sure to save the document in Microsoft Word version 2003 or older, before uploading to ScholarOne Manuscripts. When uploading this checklist to ScholarOne Manuscripts, please choose the "Supplementary Material for Review" category.

Please note that if your paper is accepted, the checklist will be published as an Online Supporting Information.

For any questions, please contact the Cytometry Part A editorial office at Cytometrya@wiley.com.

Framework Criteria	What to report	Please complete each criterion	Date	Data files and layouts
1.1 Preanalytical variables conforming to MISEV guidelines.	Preanalytical variables relating to EV sample including source, collection, isolation, storage, and any others relevant and available in the performed study.	See Methods.		
1.2 Experimental design according to MIFlowCyt guidelines.	EV-FC manuscripts should provide a brief description of the experimental aim, keywords, and variables for the performed FC experiment(s) using MIFlowCyt checklist criteria: 1.1, 1.2, and 1.3, respectively. Template found at www.evflowcytometry.org.	See Introduction, Supporting Info.		
2.1 Sample staining details	State any steps relating to the staining of samples. Along with the method used for staining, provide relevant reagent descriptions as listed in MIFlowCyt guidelines (Section 2.4 Fluorescence Reagent(s) Descriptions).	Sample staining was performed as directed in the vFC Protocol. A fresh 10x vFRed working solution was prepared from the 100x stock in Vesicle Staining Buffer. Staining reactions consisted of 5 ul diluted sample, 5 ul vFRed 10x Working Solution, and 5 uL 10x antibody added to a total volume of 50 uL in Vesicle Staining Buffer. Samples were incubated for 1 hour at ambient temperature. Following staining, sample was diluted 1000-fold for analysis.		
2.2 Sample washing details	State any steps relating to the washing of samples.	No washing was performed.		
2.3 Sample dilution details	All methods and steps relating to sample dilution.	Samples were subjected to a pre-stain dilution (10-40-fold, as determined in preliminary experiments), and a 1000-fold post stain dilution prior to measurement.		
3.1 Buffer alone controls.	State whether a buffer-only control was analyzed at the same settings and during the same experiment as the samples of interest. If utilized it is recommended that all samples be recorded for a consistent set period of time e.g. 5 minutes, rather than stopping analysis at a set recorded event count e.g. 100,000 events. This allows comparisons of total particle counts between controls and samples.	Buffer-only controls showed fewer than 1000 Vesicle-gated events per 100 uL analyzed.	5/22/20	
3.2 Buffer with reagent controls.	State whether a buffer with reagent control was analyzed at the same settings, same concentrations, and during the same experiment as the samples of interest. If used state what the results were.	Buffer plus reagent controls showed fewer than 1000 Vesicle-gated events per 100 uL analyzed. (Figure S7)	5/22/20	
3.3 Unstained controls.	State whether unstained control samples were analyzed at the same settings and during the same experiment as stained samples. If used, state what the results were, preferably in standard units.	Unstained controls were similar to Buffer-only controls and showed fewer than 1000 events per 100 uL analyzed. (Not shown)		
3.4 Isotype controls.	The use of isotype controls is applicable to immunofluorescence labelling only. State whether isotype controls were analyzed at the same settings and during the same experiment as stained samples. If utilized, state which antibody they are matched to, the concentration used, and what the results were (Section 4.2, 4.3, 4.4). Due to conjugation differences between manufacturers if should be stated if the isotype controls are from the same manufacturer as the matched antibodies.	A lack of detectable Fc Receptor mediated binding was assessed on a subset of samples using an irrelvent IgG1 at a concentration of 5 nM. The median fluorescence of the isotype-stained sample was not significantly above the background of unstained sample, indicating undetectable Fc Receptor binding. Figure S9.	5/28/20	

3.5 Single-stained controls.	State whether single-stained controls were included. If used state whether the single-stained controls were recorded using the same settings, dilutions, and during the same experiment as stained samples and state what the results were, preferably in standard units (Section 4.2, 4.3, 4.4).	Single stained controls were analyzed as part of optimization and validation of the multicolor assays.		
3.6 Procedural controls.	State whether procedural controls were included. If used, state the procedure and if the procedural controls were acquired at the same settings and during the same experiment as stained samples.	No “procedural” controls were identified.		
3.7 Serial dilutions.	State whether serial dilutions were performed on samples and note the dilution range and manner of testing. The fluorescence and/or scatter signal intensity would ideally be reported in standard units (see Section 4.3, 4.4) but arbitrary units can also be used. This data is best reported by plotting the recorded number events/concentration over a set period of time at different sample dilution. The median fluorescence intensity at each of the dilutions should also ideally be plotted on the same or a separate plot.	In preliminary experiment, serial dilutions of samples were performed on selected samples to determine an optimal sample pre-stain dilution, which showed proportional decrease in events counts with minimal change in median fluorescence over a greater than >50-fold range (from ~ 1:10 – 1:640.		
3.8. Detergent treated EV-samples	State whether samples were detergent treated to assess lability. If utilized, state what detergent was used, the end concentration of the detergent, and what the results were of the lysis.	Detergent lability was assessed on a sub-set of samples by treatment of stained sample with 0.1% Empigen BB prior to post-stain dilution and analysis. Greater than 90% of the marker-positive (TS, CFSE,antibody) events were eliminated by detergent treatment.	5/22/20	
4.1 Trigger Channel(s) and Threshold(s).	The trigger channel(s) and threshold(s) used for event detection. Preferably, the fluorescence calibration (Section 4.3) and/or scatter calibration (Section 4.4) should be used in order to report the trigger channel(s) and threshold(s) in standardized units.	Data acquisition was triggered by vFRed fluorescence (488-690/50), with a threshold (1900 arbitrary units) set to accept ~2 events/second with a buffer-only sample, and ~20 events/sec for a buffer +vFRed sample.		
4.2 Flow Rate / Volumetric quantification.	State if the flow rate was quantified/validated and if so, report the result and how they were obtained.	The sample volumetric flow rate was calibrated using counting beads (Spherotech AccuCount), and was found to be within 10% of the instrument spec of 1 uL/sec.		
4.3 Fluorescence Calibration.	State whether fluorescence calibration was implemented, and if so, report the materials and methods used, catalogue numbers, lot numbers, and supplied reference units for the standards. Fluorescence parameters may be reported in standardized units of MESF, ERF, or ABC beads. The type of regression used, and the resulting scatter plot of arbitrary data vs standard data for the reference particles should be supplied.	Fluorescence response was calibrated in units of MESF using hard-dyed nanoRainbow calibration particles (Cellarcus Biosciences) that had been cross-calibrated against FITC (Quantum FITC MESF beads, Bangs Labs #555). and PE (QuantiBrite PE beads, BD Biosciences #340495) on the sme instrument.		
4.4 Light Scatter Calibration.	State whether and how light scatter calibration was implemented. Light scatter parameters may be reported in standardized units of nm2, along with information required to reproduce the model.	No light scatter calibration was performed.		

5.1 EV diameter/surface area/volume approximation.	State whether and how EV diameter, surface area, and/or volume has been calculated using FC measurements.	EV size was estimated from the vFRed^{TM} intensity using the linear relationship between the population surface area and vFRed fluorescence distributions. Synthetic lipid vesicles with a uniform size distribution (estimated by NTA) and a lipid composition similar to a mammalian cell plasma membrane (Lipo100^{TM}, Cellarcus Biosciences, # CBS-1), were stained with vFRed^{TM} and measured by flow cytometry. The Lipo100^{TM} population diameter distribution was calculated from the NTA diameter distribution assuming a spherical geometry, and linear regression performed against the vFRed^{TM} fluorescence distribution to determine the F/nm2, which was then used to estimate the size distribution of unknowns.		
5.2 EV refractive index approximation.	State whether the EV refractive index has been approximated and how this was done.	No EV refractive index approximation was performed.		
5.3 EV epitope number approximation.	State whether EV epitope number has been approximated, and if so, how it was approximated.	Immunofluorescence intensities are presented in units of MESF, which might be considered to represent the epitope abundance to within a factor of 2, given the bivalent nature of the IgGs used.		
6.1 Completion of MIFlowCyt checklist.	Complete MIFlowCyt checklist criteria 1 to 4 using the MIFlowCyt guidelines. Template found at www.evflowcytometry.org.	Included in Supporting Information.		
6.2 Calibrated channel detection range	If fluorescence or scatter calibration has been carried out, authors should state whether the upper and lower limits of a calibrated detection channel were calculated in standardized units. This can be done by converting the arbitrary unit scale to a calibrated scaled, as discussed in Section 4.3 and 4.4, and providing the highest unit on this scale and the lowest detectable unit above the unstained population. The lowest unit at which a population is deemed 'positive' can be determined a variety of ways, including reporting the 99th percentile measurement unit of the unstained population for fluorescence. The chosen method for determining at what unit an event was deemed positive should be clearly outlined.	In general, fluorescence channels had a calibrated range of ~0-10,000 MESF/ABVs, with limits of detection (LOD, background +3SD) ranging from 20-200 MESF/ABVs).		
6.3 EV number/concentration.	State whether EV number/concentration has been reported. If calculated, it is preferable to report EV number/concentration in a standardized manner, stating the number/concentration between a set detection range.	EV concentrations are reported in EVs/mL, which is calculated from the number of events detected in the Vesicles gate and accounting for the volume analyzed and all pre- and post-stain dilutions. Marker-positive events we calculated from the number of events exceeding an arbitrary gate set at the ~99.5 percentile of the negative population.		
6.4 EV brightness.	When applicable, state the method by which the brightness of EVs is reported in standardized units of scatter and/or fluorescence.	EV brightness is reported in arbitrary units, and as MESF/ABV units where possible and appropriate.		
7.1. Sharing of data to a public repository.	Provide a link to the experimental data in a public data repository.	Data have been uploaded to the ISAC Flow Repository.		
