# Supplementary material for: Generation and Application of a Reporter Cell Line for the Quantitative Screen of Extracellular Vesicle Release
Source: Front Pharmacol. 2021 Apr 16;12:668609. doi: 10.3389/fphar.2021.668609 (PMC8085554; doi:10.3389/fphar.2021.668609)
Supplement: Supplementary file 2 [file datasheet2.pdf]

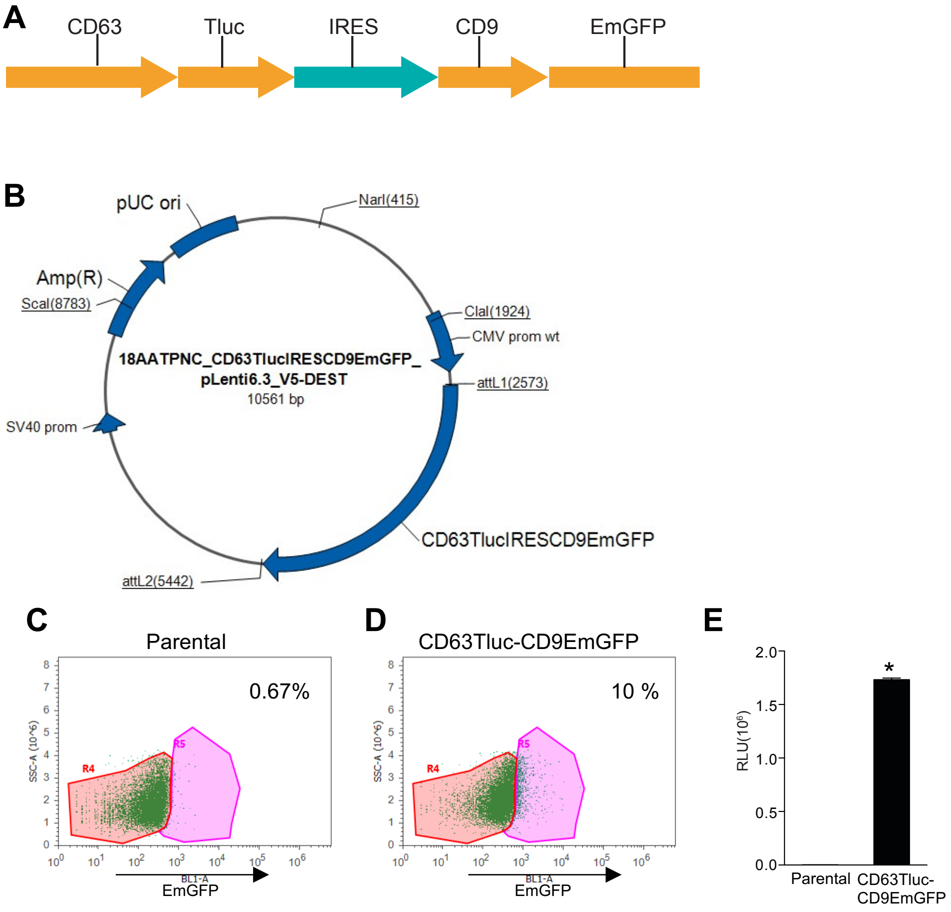

Supplementary Figure 1. Plasmid map of CD63Tluc-CD9EmGFP pLenti6.3 V5-DEST. (**A**) The synthetic gene CD63TlucIRESCD9EmGFP was assembled from synthetic oligonucleotides and PCR products. (**B**) The fragment was inserted into pLenti6.3_V5-DEST_A244. The plasmid DNA was purified from transformed bacteria and concentration determined by UV spectroscopy. The final construct was verified by sequencing. The sequence identity within the insertion sites was 100%. (**C** and **D**) CD63Tluc-CD9EmGFP transduced THP-1 cells were analyzed on an Attune flow cytometer for fluorescence. Approximately 10% of the cells were fluorescent in the FL-1 channel. (**E**) The pool of stably transduced THP-1 cells had significantly higher levels of relative luciferase activity (RLU) compared to the parental cell line. * denotes $p<0.01$ by Student's *t*-test.

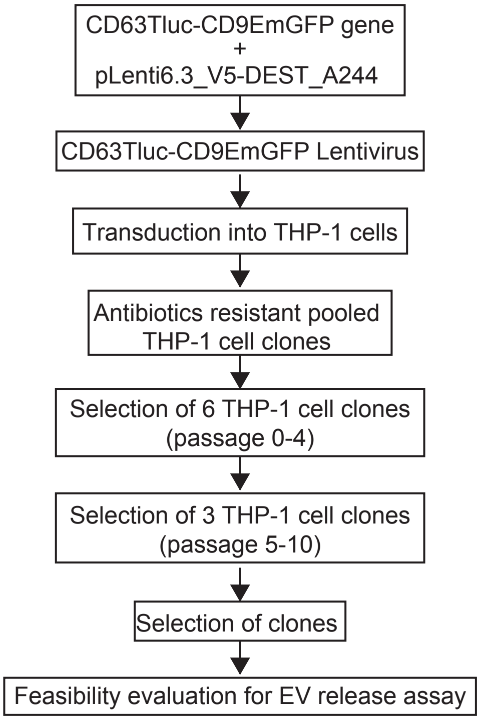

Supplementary Figure 2. Schematic of reporter cell line generation for quantitative measurement of EV release.

A CD63Tluc-CD9EmGFP gene fragment was cloned under a CMV promoter in a lentivirus vector, pLenti6.3_V5-DEST_A244. The construct was transduced into THP-1 cells, and a stably expressing cell-pool was expanded with antibiotic-selection. Six clones were tested for CD63Tluc and CD9EmGFP expression. Three clones showing high levels of Tluc expression were further expanded, and one clone was selected for further characterization by EV release assays.

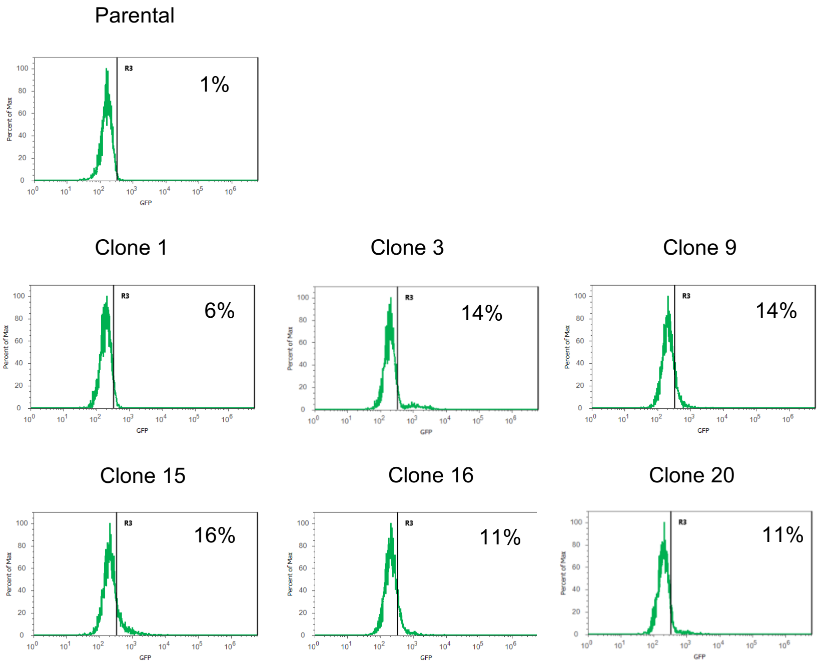

Supplementary Figure 3. Significant reduction of EmGFP expressing cells after the 4th passage. CD63Tluc-CD9EmGFP transduced THP-1 clones were selected on the basis of FL-1 fluorescence. After passage 4 the cloned populations were examined by flow cytometric analysis. Data presented are GFP intensity per % Max of cell count. R3 gate represents percent GFP positive cells.

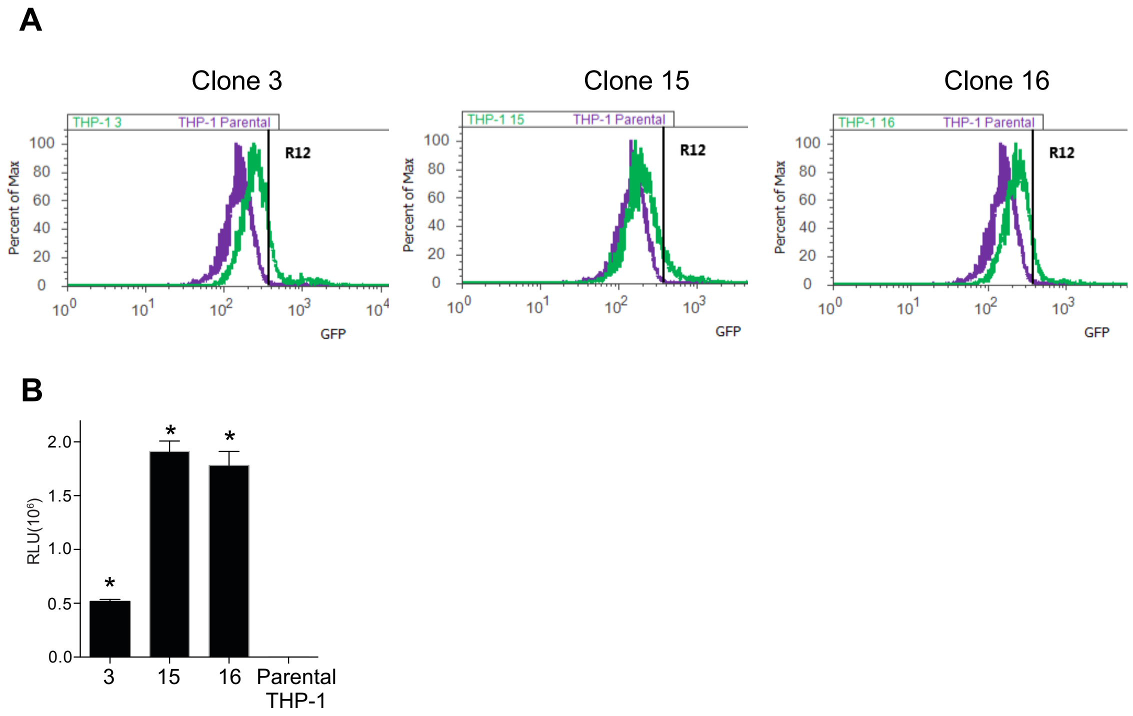

Supplementary Figure 4. Luciferase activity and EmGFP expression of selected clones. At passage 10, the cells were analyzed for GFP expression by flow cytometry (**A**) and for luciferase expression using TLuc assay (**B**). (**A**) GFP fluorescence levels in all the three clones tested were higher than the non-transduced cells and were stable after passage 4. Data presented as GFP intensity per % Max of cell count. (**B**) The cloned cells were seeded at 50,000 cells per well. Compared to the parental THP-1 cells, all three clones showed higher luciferase activity. Data shown are means ± SD. *P<0.001 by ANOVA with Dunns post hoc comparison to the parental line.

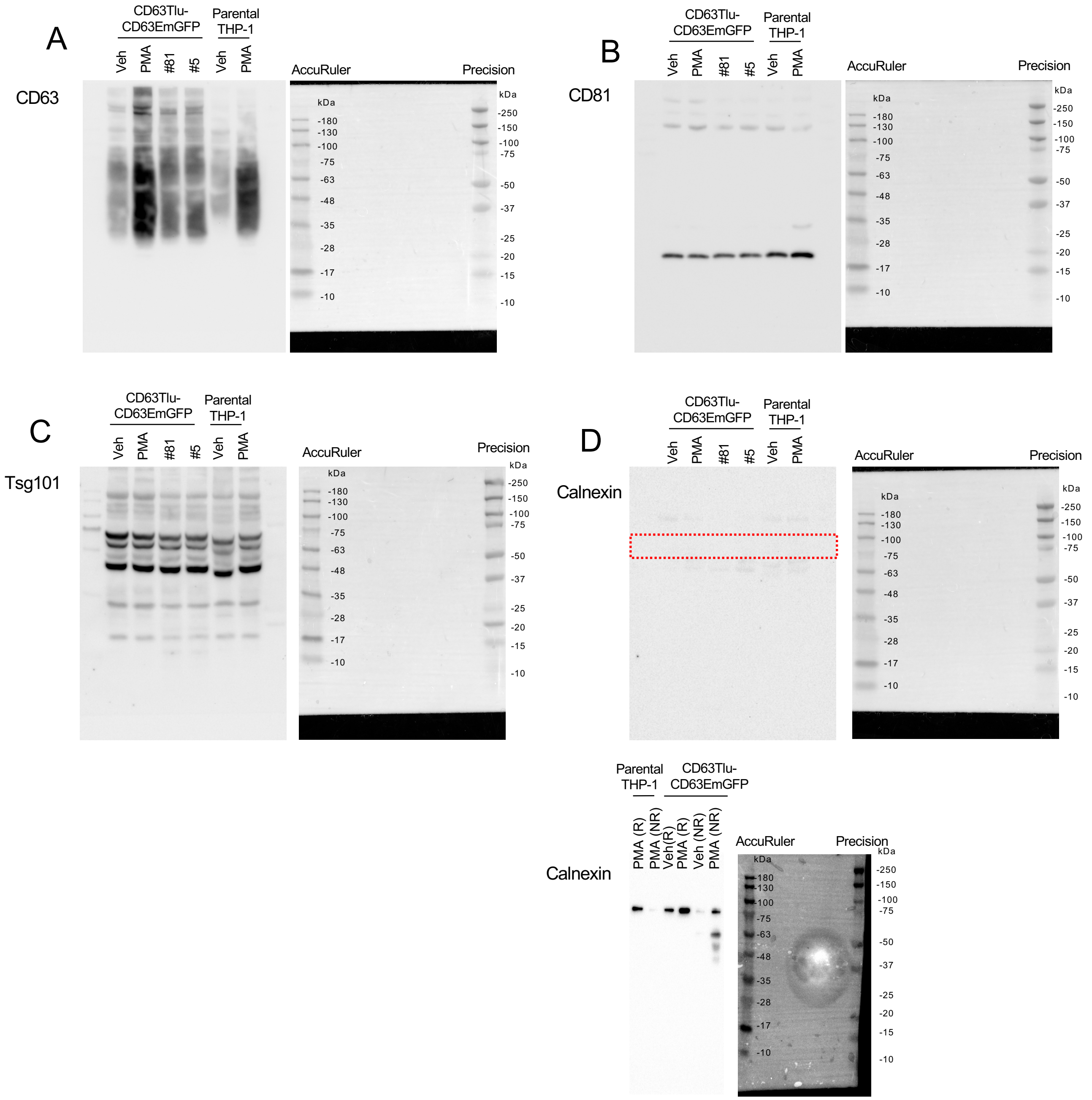

Supplementary Figure 5. Unmodified Western blot scans from Figure 5. The blots were stained with anti-CD63 (**A**), anti-CD81 (**B**), anti-Tsg101(**C**), and anti calnexin (**D**). In some experiments, EVs samples were run under reducing (R) or non-reducing (NR) conditions. Two molecular weight markers (Precision Plus Protein™ Dual Color Standards, and AccuRuler Prestained Protein Ladder) were used. The red-dotted box indicates the molecular weight range where calnexin is expected to be detected (~80kDa).

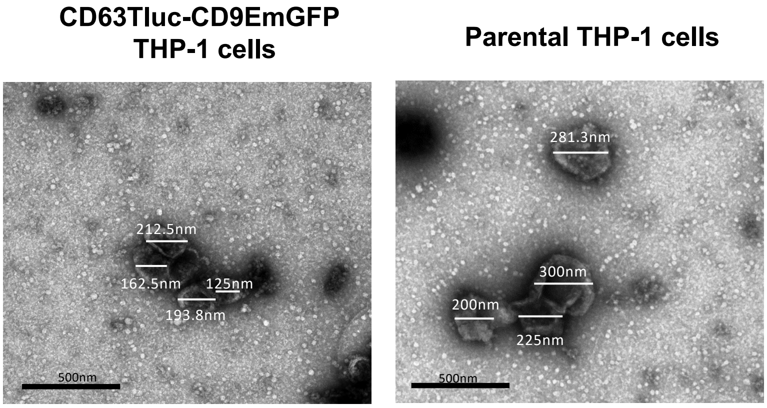

Supplementary Figure 6. The size of EVs measured by TEM images. CD63Tluc-CD9EmGFP reporter cells or parental THP-1 cells were incubated with vehicle (0.1% DMSO) for 48 h and EVs were isolated using ExoQuick TC. The diameter of EVs were measured and calculated using the scale bars (500nm).

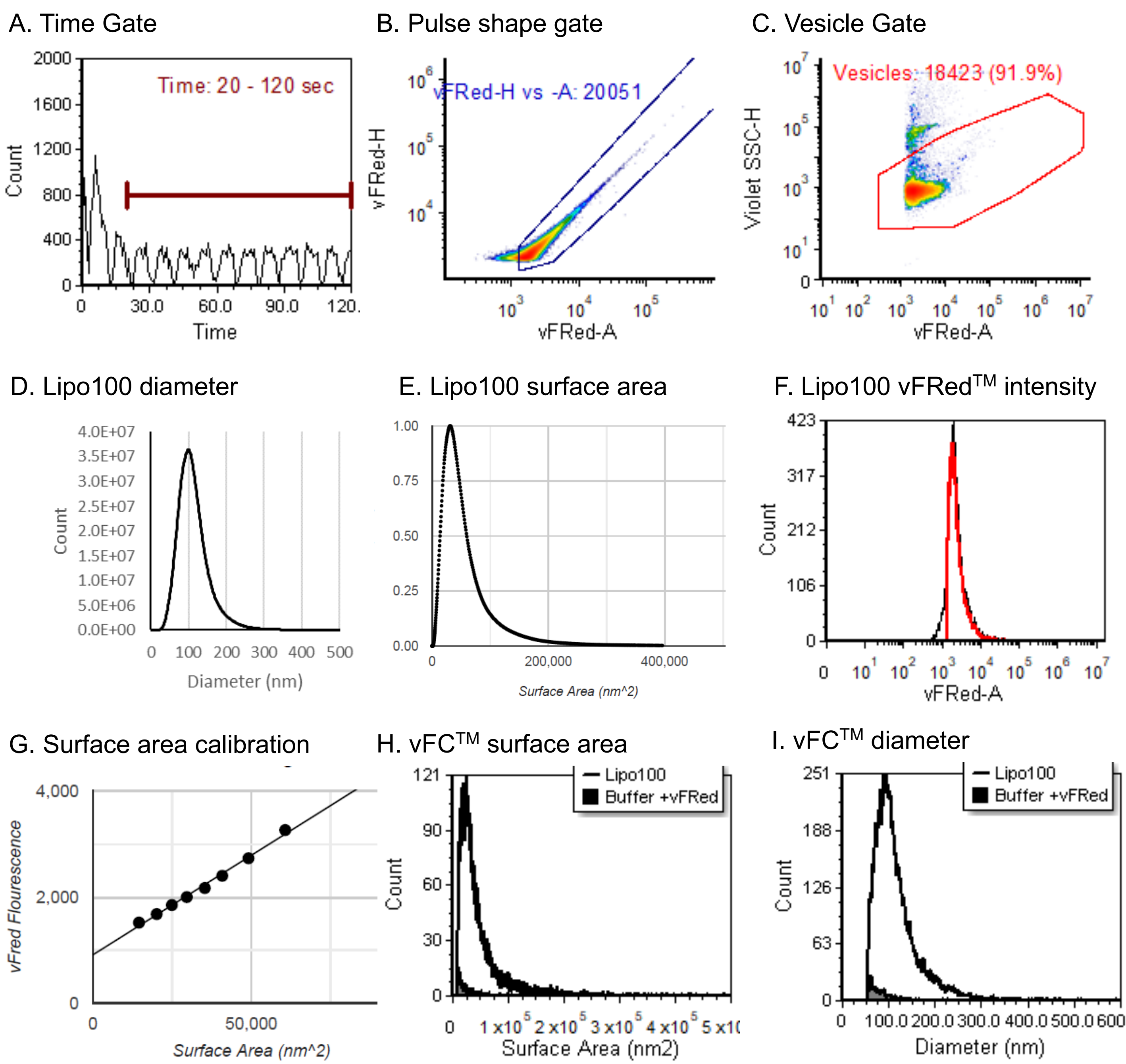

Supplementary Figure 7. vFC^{TM} gating and vesicle size calibration. (**A-C**) vFC^{TM} gating includes a Time Gate (**A**) to eliminate background from fluidic disturbances, a Pulse Shape Gate (**B**), which selects events with characteristic vFRed^{TM} signal pulse height and area that can eliminate low intensity background events, and a Vesicle Gate (**C**), which selects events with characteristic vFRed^{TM} and VSSC intensities. (**D-I**) Vesicle size calibration involves a synthetic vesicle standard (Lipo100^{TM}) with a measured Diameter distribution measured by NTA (**D**) and from which the Surface area distribution is calculated **(E).** The Fluorescence distribution of vFRed-stained Lipo100^{TM} (**F**) is proportional to the surface area distribution (**G**) and linear regression produces a line with a slope of F/nm2, which allows calibration of the vFC^{TM} Surface Area (**H**) and Diameter (**I**) scales. Buffer +vFRed^{TM} controls showed negligible background.

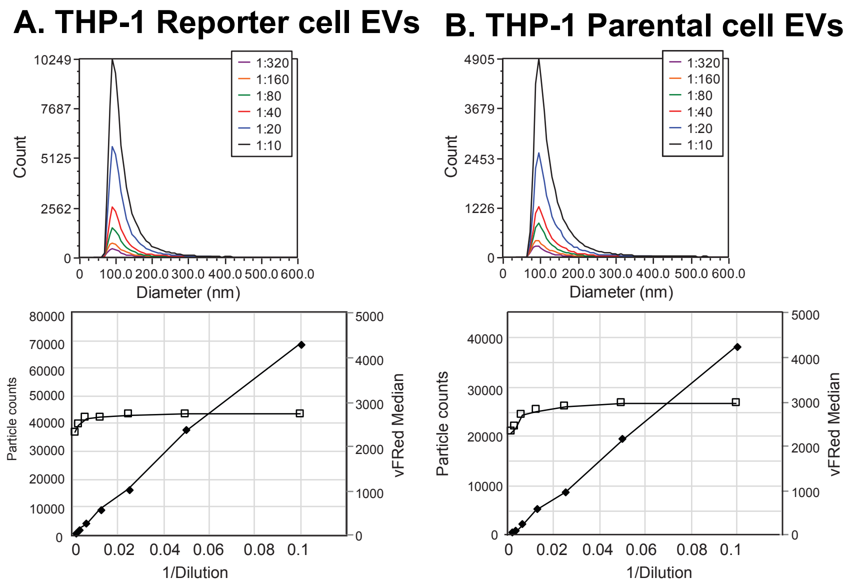

Supplementary Figure 8. Demonstration of single particle events by dilution of stained samples. EVs from CD63Tluc-CD9EmGFP reporter cells (**A**) and parental THP-1 cells (**B)** were stained with vFRed and serially diluted. Upper panels show histograms of the measured events. Lower panels show single particle events per µL of sample and median fluorescence intensity of vFRed. The event rate decreased in proportion to the dilution, but the median fluorescence did not change, consistent with the analysis of single particles.

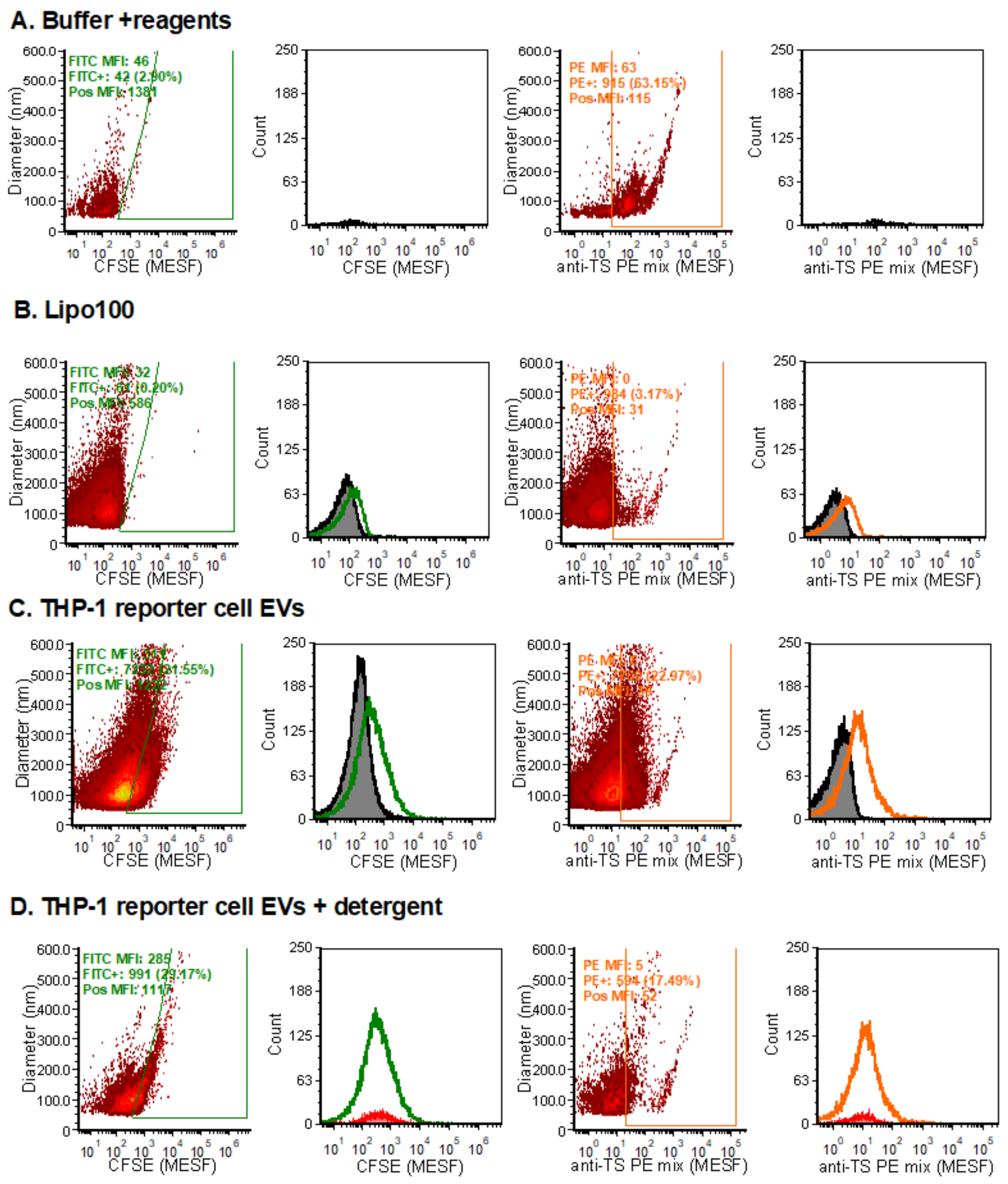

Supplementary Figure 9. vFC^{TM} immunofluorescence controls. (**A**) Buffer + reagent controls measure the background events associated with buffers and reagents. (**B**) Lipo100 serves as a negative control for staining by CFSE and fluorescent antibodies to show low levels of background staining compared to unstained vesicles (shaded). (**C**) The THP-1 reporter cell EVs show staining by CFSE and a mixture of PE-labeled anti-TS antibodies (CD9/63/81) compared to unstained EVs (shaded). (**D**) Detergent treatment (red shaded) demonstrates that >90% of detected events are detergent-sensitive

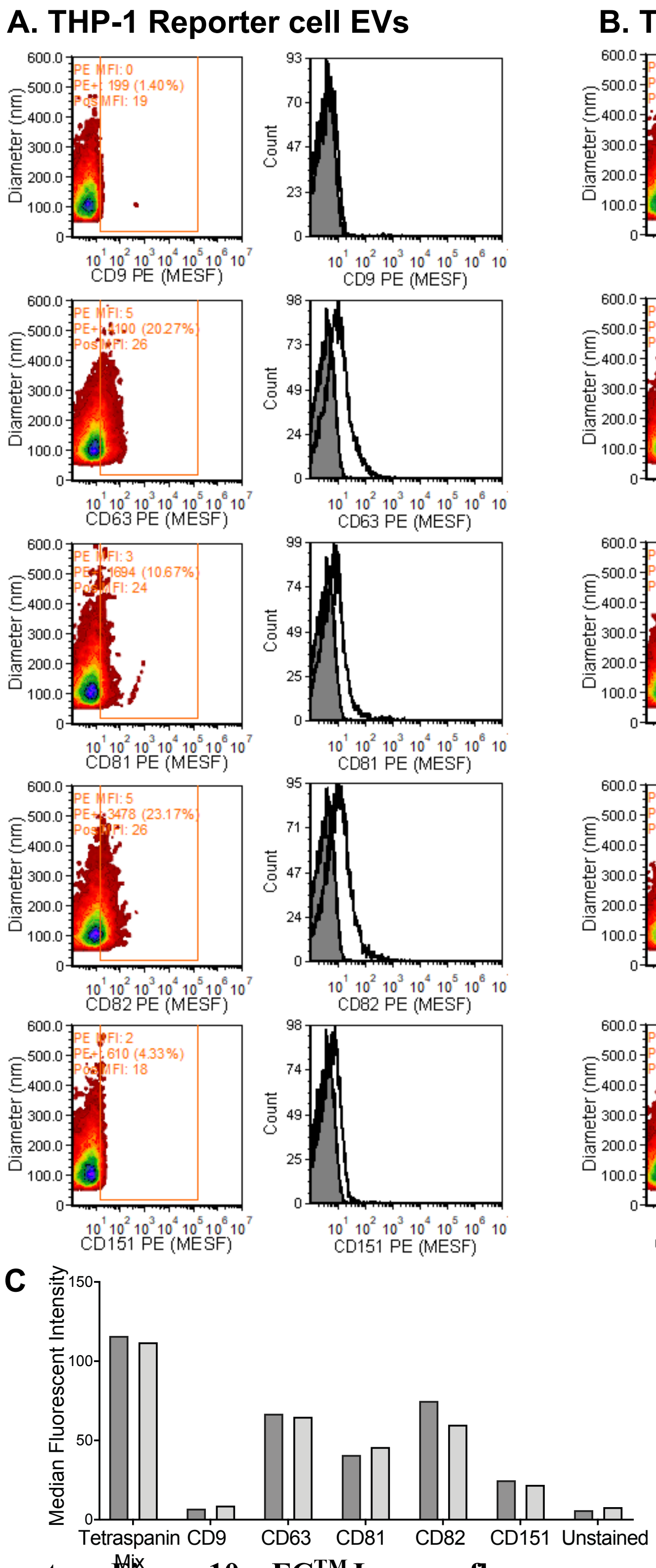

Supplementary Figure 10. vFC^{TM} Immunofluorescence of tetraspanin expression on EVs from CD63Tluc-CD9EmGFP reporter cells (A) and parental THP-1 cells (B). Presented are two parameter histograms of EV size vs immunofluorescence intensity and single parameter histograms overlays of immunofluorescence of stained and unstained (shaded) cells. Note that all antibodies are IgG1 isotypes, and the lack of detectable CD9 staining also serves as an isotype control to demonstrate the lack of detectable Fc receptor mediated IgG binding on these EVs. (**C**) EV cargo of the reporter and parental cells were compared. The samples were stained with vFRed, CFSE, and one of the following EV cargo markers. PE-conjugated anti-TS mix, CD9, CD63, CD81, CD82, or CD151 and analyzed by CytoFLEX S.

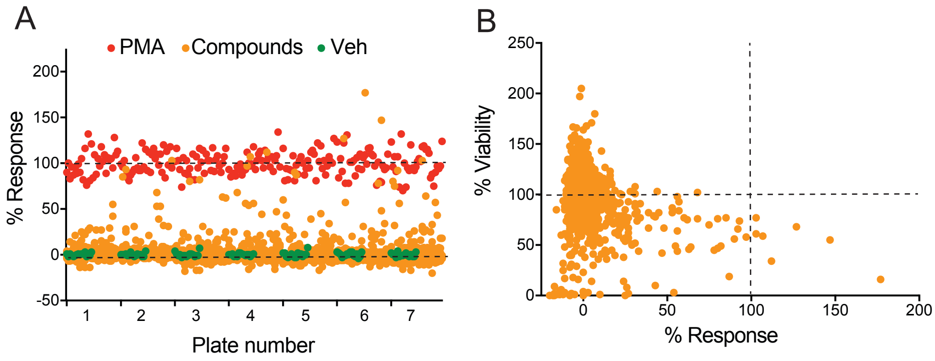

C

Compound ID	% Response ratio	% Viability
#248	326 ± 16	12.0 ± 0.2
#246	106 ± 20	27.4 ± 0.3
#253	93 ± 8	34.7 ± 1
#244	68 ± 6	55.5 ± 0.8
#247	60 ± 13	26.6 ± 0.2

Supplementary Figure 11. HTS using CD63Tlu-CD9EmGFP reporter cells (**A**) Example of the Tluc activity distribution of the screen (day 2). Orange circle, test compounds; red circle, PMA; and green circle, vehicle control. The response ratio was calculated using the following formula; % response=100 × (compound RLU-vehicle RLU)/(PMA RLU-vehicle RLU). (**B**) The % viability (day 2) negatively correlated with Tluc activities in CD63 reporter cells. (**C**) % response and % viability of top 5 compounds are presented.

Supplementary Table 1. Raw data for Table 1 (Tluc activity in CD63TlucCD9EmGFP reporter cells and culture supernatants)

	Cells (RLU)		Supernatant (RLU)		% release	
Vehicle						
exp 1	15919		1028		6.5%	
exp 2	26016		1032		4.0%	
exp 3	51668		956		1.9%	
exp 4	55644		1105		2.0%	
mean, SD	37312	± 19386	1030	± 61	3.6%	± 2.2%
LPS (10ng/ml)						
exp 1	60978		1946		3.2%	
exp 2	59007		1865		3.2%	
exp 3	260771		2831		1.1%	
exp 4	174855		3700		2.1%	
mean, SD	138903	± 97639	2586	± 862	2.4%	± 1.0%
PMA (50ng/ml)						
exp 1	128843		2797		2.2%	
exp 2	169204		4262		2.5%	
exp 3	202397		3812		1.9%	
exp 4	304463		3786		1.2%	
mean, SD	201227	± 75109	3664	± 618	2.0%	± 0.5%
#5 (5μM)						
exp 1	66397		2870		4.3%	
exp 2	71850		3602		5.0%	
exp 3	119263		3085		2.6%	
exp 4	87328		2940		3.4%	
mean, SD	86210	± 23752	3124	± 331	3.8%	± 1.1%
#81 (5μM)						
exp 1	75701		3202		4.2%	
exp 2	72404		3932		5.4%	
exp 3	112308		3211		2.9%	
exp 4	81510		3009		3.7%	
mean, SD	85481	± 18277	3339	± 407	4.1%	± 1.1%

a) Data from 4 independent experiments were presented.
b) % release =luciferase activity of supernatant/(Tluc activities of cells + supernatant)
